# A mixed methods study to assess the impact of COVID-19 on maternal, newborn, child health and nutrition in fragile and conflict-affected settings

**DOI:** 10.1186/s13031-022-00465-x

**Published:** 2022-06-03

**Authors:** Mariana Rodo, Lucy Singh, Neal Russell, Neha S. Singh

**Affiliations:** 1 Health in Humanitarian Crises Centre, London School of Hygiene and Tropical Medicine, London, UK; 2 Independent Consultant, London, UK

**Keywords:** COVID-19, Maternal health, Newborn health, Child health, Conflict, Refugees, Emergencies

## Abstract

**Background:**

The impacts of COVID-19 are unprecedented globally. The pandemic is reversing decades of progress in maternal, newborn, child health and nutrition (MNCHN), especially fragile and conflict-affected settings (FCAS) whose populations were already facing challenges in accessing basic health and nutrition services. This study aimed to investigate the collateral impact of COVID-19 on funding, services and MNCHN outcomes in FCAS, as well as adaptations used in the field to continue activities.

**Methods:**

A scoping review of peer-reviewed and grey literature published between 1st March 2020–31st January 2021 was conducted. We analysed 103 publications using a narrative synthesis approach. 39 remote semi-structured key informant interviews with humanitarian actors and donor staff within 12 FCAS were conducted between October 2020 and February 2021. Thematic analysis was undertaken independently by two researchers on interview transcripts and supporting documents provided by key informants, and triangulated with literature review findings.

**Results:**

Funding for MNCHN has been reduced or suspended with increase in cost of continuing the same activities, and diversion of MNCHN funding to COVID-19 activities. Disruption in supply and demand of interventions was reported across different settings which, despite data evidence still being missing, points towards likely increased maternal and child morbidity and mortality. Some positive adaptations including use of technology and decentralisation of services have been reported, however overall adaptation strategies have been insufficient to equitably meet additional challenges posed by the pandemic, and have not been evaluated for their effectiveness.

**Conclusions:**

COVID-19 is further exacerbating negative women’s and children’s health outcomes in FCAS. Increased funding is urgently required to re-establish MNCHN activities which have been deprioritised or halted. Improved planning to sustain routine health services and enable surge planning for emergencies with focus on the community/service users throughout adaptations is vital for improved MNCHN outcomes in FCAS.

**Supplementary Information:**

The online version contains supplementary material available at 10.1186/s13031-022-00465-x.

## Introduction

It is estimated that 274 million people will need humanitarian assistance and protection in 2022, of which women and children make up the majority [1]. COVID-19 has had unprecedented health, social and economic impacts globally. It has exacerbated pre-existing inequalities in maternal, newborn, and child health (MNCH) outcomes, with fragile and conflict-affected settings (FCAS) particularly at risk given that these settings reported considerably worse health outcomes compared to other contexts even before the pandemic [2]. This is in large part due to the fragility of the health systems in FCAS, which are operating with restricted resources, decreased numbers of healthcare workers, interrupted supplies and damaged health facilities [3]. The impacts of the pandemic in FCAS are also disproportionately affecting women and children due to existing factors of vulnerability including lack of decision-making power, the gendered role of women as caregivers increasing their risk of virus exposure, and exacerbation of sexual and gender-based violence [4]. Yet, whilst women’s and children’s health and nutrition needs are increasing, essential services for them are now even more difficult to access and utilise, and existing health needs and humanitarian crises have become “secondary” due to the pandemic as COVID-19 measures are prioritised over maintaining essential health services [5].

Evaluations of humanitarian responses to previous crises including the Ebola epidemic in the Democratic Republic of Congo (DRC) and West Africa and the cholera outbreak in Yemen have provided numerous lessons and insights into the impact of outbreaks on health outcomes for vulnerable populations in FCAS [6–8]. Evidence from previous outbreaks clearly demonstrates that maintaining essential health care services is critical to reduce additional deaths and that the indirect effects of outbreaks and epidemics exceed the mortality and morbidity caused by it [7, 9]. This is particularly evident in contexts without resilient health systems, such as FCAS. Previous research evaluating indirect crises-related maternal and neonatal deaths in Sierra Leone during the Ebola epidemic found large reductions in antenatal care, family planning, facility delivery and postnatal care with estimates of at least 3600 additional maternal, neonatal and stillbirth deaths in the year 2014–2015 [8].

In previous epidemics in FCAS such as Ebola and Zika, there has been a diversion of funding and other resources (such as staff and supplies) away from MNCH and towards the health emergency presented by the epidemic [10]. This has implications for MNCH service provision and outcomes. In previous epidemics, it resulted in increased stillbirth, maternal and neonatal mortality, as well as compromising quality of care such as with poor infection control practices leading to nosocomial Ebola infection of pregnant women. Additionally, poor quality of care was a deterrent to attendance at MNCH healthcare facilities [10]. There is evidence to suggest funding diversions are occurring in the COVID-19 pandemic away from routine and essential healthcare and towards pandemic response in several humanitarian settings [5]. Whilst this publication is focussed on non-communicable diseases, it raises concerns for funding in MNCHN—particularly in the context of MNCH funding diversions seen in prior epidemics.

There have been a number of studies to date assessing the impact of the COVID-19 pandemic on maternal, newborn, child health and nutrition (MNCHN) outcomes and service utilisation globally demonstrating adverse indirect impacts including increased sexual and gender-based violence, reduced prenatal visits, overwhelmed healthcare infrastructure and implementation of potentially harmful policies despite little evidence [11–14]. However, no studies to date have focused exclusively on FCAS. To our knowledge, ours is the first study to document the impact of COVID-19 on MNCHN outcomes, funding and programming in FCAS as well as documenting adaptations used in these settings. Our study aims to take an exploratory and explanatory approach to examine this and highlight areas for further research. It intends to provide reflections for donors, humanitarian actors and others working in this sector to improve policy and programming for women and children during and beyond the COVID-19 pandemic.

## Methods

This study used mixed methods drawing on two data sources: (1) a scoping literature review and (2) key informant interviews with donor staff and humanitarian actors working in FCAS. The approach was both exploratory and explanatory. Each data source was used to generate new evidence and both sources were triangulated to validate findings.

### Literature review

#### Search strategy

Our review included published peer-reviewed and grey literature both publicly available and shared by the key informants during or after the interviews took place. The search included publications from 1 March 2020 to 31 January 2021 to account for the known start of the pandemic in FCAS. The search strategy (Additional file 1) was based on a published search protocol for a study on a similar topic in low- and middle-income countries (LMICs) [15]. The search terms used referred to categories of population (maternal, newborn, child and adolescent), health services (health care, health systems and provision/use) and settings (FCAS and relevant LMICs).

We searched for publications in Embase, Medline and Global Health databases using the Ovid^®^ search interface. This search was complemented by screening the Repository composed by John Hopkins Centre for Humanitarian Health for relevant results until 2 February 2021. We also screened reference lists of relevant articles including a global scoping review on maternal and perinatal health [13] to identify additional publications.

Grey literature was searched in publicly available records from Interagency Working Group for Reproductive Health in Crises, Save the Children, Médecins Sans Frontières (MSF), the International Rescue Committee (IRC), CARE International, International Committee of the Red Cross, READY Initiative, United Nations Children's Fund (UNICEF), United Nations Population Fund (UNFPA), United Nations High Commissioner for Refugees, Healthy Newborn Network, Women’s Refugee Commission, Marie Stopes international and the Global Financing Facility. We searched for publications related to current UK Government work on the UK Parliament website. We included articles published in academic journals (such as editorials, commentaries, and perspective pieces), reports, relevant news articles, pre-prints of studies and information shared by the interviewees. In addition, we included the available data on the UNICEF’s ‘Tracking the situation of children during COVID-19’ dashboard (‘UNICEF dashboard’ hereafter) [16], using the existing filter for countries with ‘other ongoing humanitarian response’.

#### Study selection and data extraction

Full inclusion and exclusion criteria for the literature review is presented in Table 1.

**Table 1:** Inclusion and exclusion criteria for the literature review

Category	Included	Excluded
Population of interest	Women, adolescents, newborns, and children living in FCAS, as defined by the World Bank [17], and in other relevant LMIC settings, such as Cox’s Bazar (Bangladesh)	Women, adolescents, newborns, and children living in other settings not included in the list from World Bank or in LMIC settings not relevant to FCAS
Intervention	Any interventions relating to MNCH and Nutrition	Publications focusing on other health areas, on clinical aspects of COVID-19 in MNCH, and sexual and reproductive health (SRH) services
Outcome	Indicators that report on the indirect impact of COVID-19 in MNCHN services	Indicators that do not report on the indirect impact in MNCHN or are only focused on the direct outcomes of COVID-19
Situation	Publications referring to the effect of the pandemic in the FCAS	Publications from prior the pandemic in the FCAS
Type of publication	Any type of publication; peer-reviewed studies and other types of publications in a journal (e.g., editorials, comments); grey literature (including pre-prints, relevant news articles and data shared by the interviewees)	
Publication date	1st March 2020–31st January 2021	Any publication published before 1st March 2020 and after 31st January 2021
Language	English, Portuguese, Spanish, French	Other languages

The population of interest for the literature review included women, adolescents, newborns and children in fragile and conflict affected states as defined by the World Bank [17] (Additional file 2). Publications from LMICs that were deemed relevant to FCAS were also included. This search focused on interventions including antenatal care (ANC), postnatal care (PNC), deliveries, essential newborn health interventions, child health interventions, vaccinations, and nutrition activities. For the outcome of interest, we excluded the direct effect of the COVID-19 on the interest population as our focus was not on the health outcome of people with COVID-19, but on the health outcomes from the overall disruption of these specialised health care services.

**Fig. 1:**
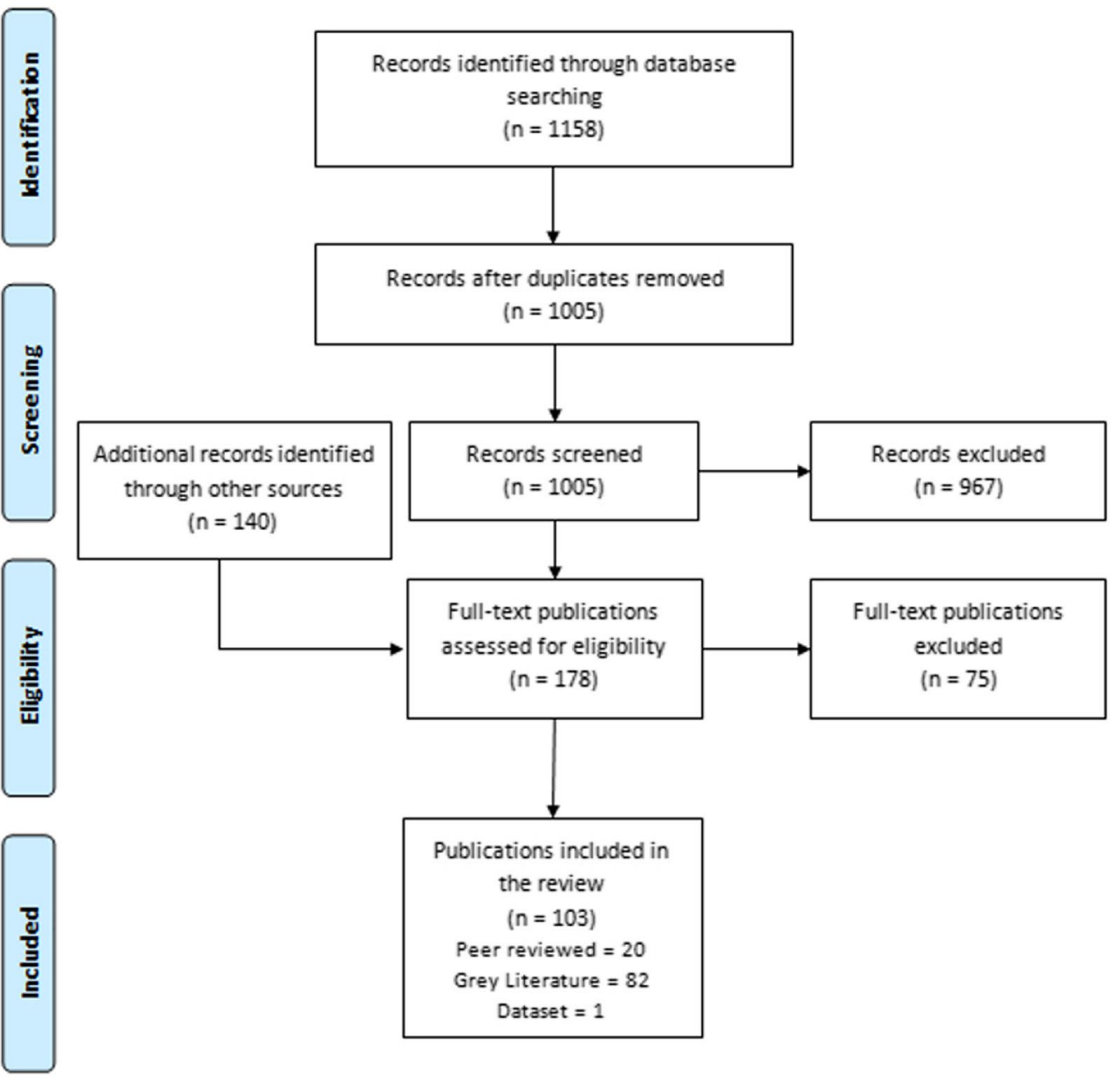
PRISMA flow diagram for publications included in the literature review

We downloaded all returned citations from databases and grey literature searches into an Endnote library and applied a standard data-screening process (Fig. 1). MR and NSS independently conducted double screening. Titles and abstracts were screened for relevance, and full-text articles screened against inclusion criteria. Grey literature publications were screened first by title and then full-text to consider inclusion. Data from the final selected studies were then extracted into a Microsoft Excel database, with data extraction fields including author and year, country, population, publication type, general topic addressed, types of services provided, and summary of publication where details on reported adaptations, funding and MNCHN outcomes were extracted. We used a narrative synthesis approach due to the heterogeneity of publication outcomes, interventions, and methods.

### Qualitative interviews

For the qualitative approach we aimed to include a broad range of humanitarian actors to attempt to collect diverse information from a range of FCAS. The inclusion criteria for key informants were humanitarian actors working in FCAS [defined as programme staff, policymakers, practitioners from local and international non-governmental organisations (NGOs) and United Nations (UN) agencies] engaged in MNCHN programming in FCAS during COVID-19 and donor staff funding MNCHN programming in FCAS. Participants were purposively recruited from a list of contacts gathered by the authors that combined information obtained from the authors’ own network, the COVID-19 Humanitarian Platform [18], and contacts provided by the Unicef team who supported this study. This was followed by snowball sampling to identify additional relevant respondents. We sent out invitation emails for recruited potential informants and interviews scheduled with the ones that accepted. All participants gave oral consent to be interviewed and to be recorded.

We conducted 39 remote semi-structured interviews from October 2020 to February 2021. The interviews were carried out via an agreed online platform. We used an adapted version of the interview guide used by the COVID-19 Humanitarian Platform [18] to collect data on programmatic adaptations and innovations in humanitarian settings during the pandemic (Additional file 3). Our guide was adapted for donor staff to include a focus on levels and trends in funding for MNCHN programmes in FCAS before and during COVID-19. All interviews were conducted in English and lasted approximately 1 h. Interviews were transcribed using the software Otter.ai^®^.

Thematic analysis was undertaken on interview transcripts and supporting documents such as internal non-published reports provided by key informants using Braun and Clarke’s six phases of thematic analysis [19]. Analysis was undertaken independently by two researchers (LS and NSS) using NVivo 12 software. Following familiarisation with the data through reading of transcripts and documents, initial codes were derived which were then collated into themes.

The interview and literature data were triangulated in an iterative process using an exploratory and explanatory approach, with cross-checking of themes across the interview and literature data prior to defining and expanding on themes.

## Results

### Characteristics of included publications and key informants

A total of 1158 citations were returned from peer-reviewed databases, with 140 additional studies identified in reference lists (Fig. 1). Following full-text screening, 103 publications met the inclusion criteria (Table 2), comprising of 20 peer-reviewed publications (Additional file 4) [20–39], 82 grey literature publications (Additional file 4) [40–121], and one dataset [16]. The peer-reviewed papers included 8 cross-sectional studies [20, 22, 24, 25, 28, 33, 34, 36], 3 qualitative [29, 37, 39], 4 modelling studies [23, 26, 27, 35] and five reviews [21, 30–32, 38]; and the grey literature included 32 non-peer reviewed articles published in an academic journal (e.g., editorials, comment, letters to editor) [40–70, 80], three pre-print studies [71, 95, 120], one blog [111], one non-peer reviewed review [72], 31 reports/briefs from humanitarian actors [75, 77–79, 81–84, 86, 91, 94, 96–99, 101, 102, 104–106, 108, 112, 114–119, 121, 122], 10 news articles [73, 74, 87, 88, 100, 107, 109, 110, 113, 123] and four presentations shared by key informants [85, 92, 93, 103]. Overall themes in the publications, 38 mentioned maternal health, 11 on newborn health, 46 in child health and 34 in nutrition. The included dataset was retrieved from the UNICEF dashboard [16] which reported data from 26 countries, of which 23 are part of the World Bank fragile and conflict affected states list [17].

**Table 2:** Overview of included publications by setting and type of publication

	Peer-reviewed literature	Grey literature	Dataset	Total
Fragile and conflict-affected setting (FCAS)	10	56	1	67
Low- and middle-income country (LMIC)	10	26	0	35
Total	20	82	1	103

**Fig. 2:**
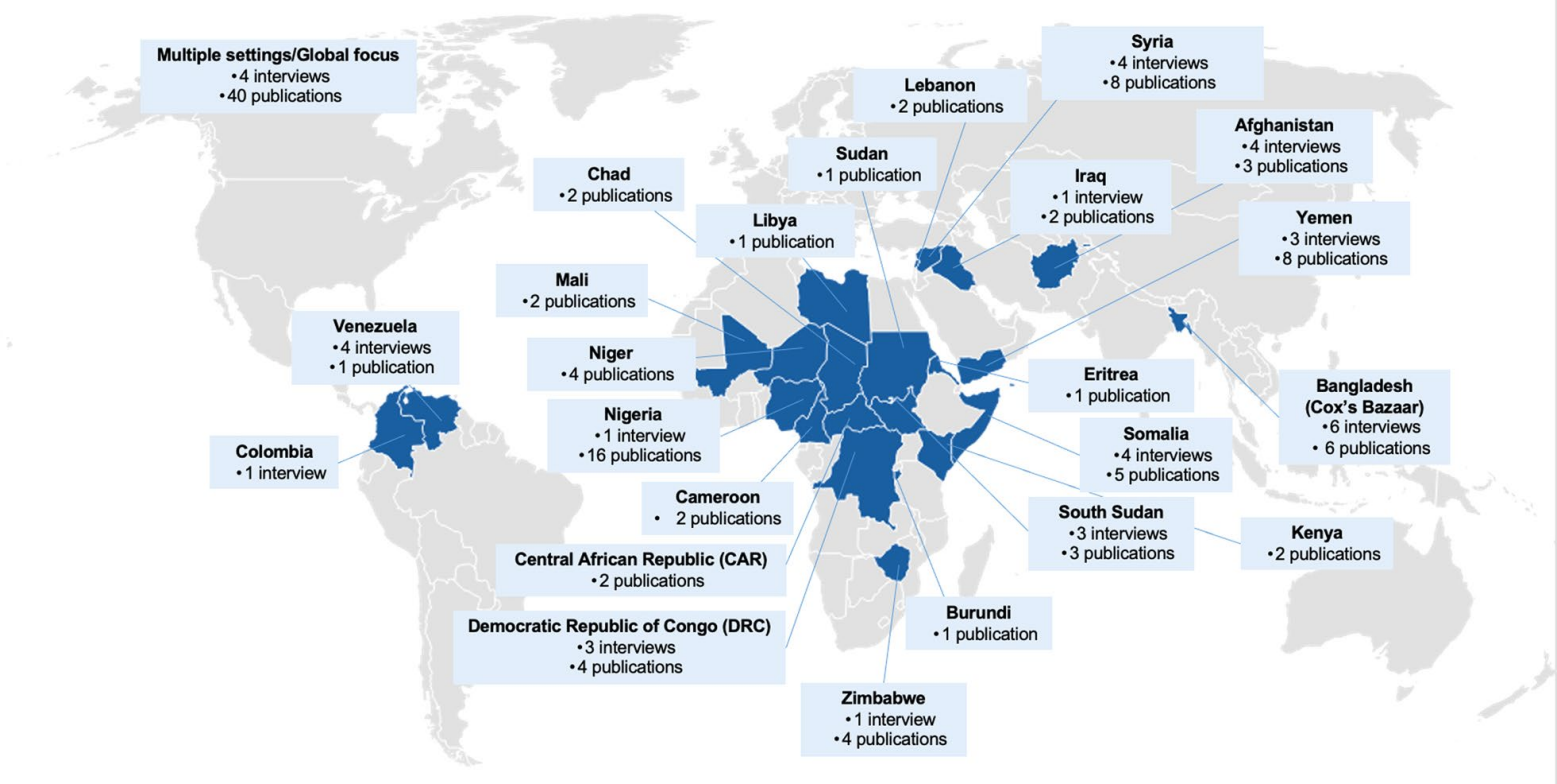
Number of included publications and interviews by country

Key informants in the study (n = 39) represented donor staff (n = 3), academics (n = 2) and humanitarian agencies (n = 34). Key informants reported findings from the following 12 fragile and conflict affected states: Afghanistan, Colombia, DRC, Iraq, Nigeria, Somalia, South Sudan, Syria, Venezuela, Yemen, Zimbabwe, and Bangladesh (Cox’s Bazaar) (Fig. 2).

The research findings are presented by the pandemic’s impact on funding for MNCHN, disruptions to these health and nutrition services, impact on MNCHN outcomes, and reported adaptation strategies for these services during the pandemic.

### Impacts of funding for MNCH and nutrition

We identified significant impacts on funding for MNCHN in FCAS. There was a clear political priority placed on COVID-19 activities in many countries from the start of the pandemic. Decisions about funding diversions away from MNCHN and towards COVID-19 specific case management activities were driven by governments, donors and stakeholders [53, 70]. Respondents highlighted that in many cases governments explicitly requested MNCHN activities to be deprioritised or prevented them from occurring in favour of COVID-19 activities.

#### Increase in cost of continuing the same activities

Three interview respondents reporting on Somalia, Iraq and Sudan and two publications [25, 66] highlighted that COVID-19 has led to significantly increased costs of maintaining existing activities for MNCHN. Data from Nigeria that considered the changes in expenditure pre- and during the pandemic found that the cost of giving birth doubled or tripled depending on the route of delivery, with personal protective equipment (PPE) accounting for a large proportion of the cost increase and COVID-19 Polymerase Chain Reaction (PCR) testing that, in many cases, was passed on to women and families [25, 66]. These findings were echoed by interview respondents who also cited increased requirements for consumables such as PPE and service modifications for infection prevention control (IPC). More money was also required for human resources to maintain existing services. This is because a significant number of existing staff with underlying medical risk factors had to be moved away from frontline roles and contexts with difficult medical evacuation, and with staff having to self-isolate or quarantine.

*“What we saw in the project, like we make a plan on usually twice a year in terms of like what the project should do and what the focus will be, and which areas need more funding or more budget, so to say, and then it gets approved by headquarters or it will have to be adjusted. So in general the plan of course made before COVID came and then basically when Corona came [NGO] had a big problem all of a sudden because it started being something which was affecting all the projects all across the world, and giving a lot of actually higher needs in terms of budgeting because of all the PPE and like all the problems concerning flights.”—International NGO, South Sudan*

#### Diversion of funding from MNCHN activities to COVID-19 related activities

The diversion of focus towards the pandemic has led to redirection of funds away from MNCHN services according to two publications and 14 interview respondents [62, 117] and 14 interview respondents reporting on Nigeria, Venezuela, Somalia, Afghanistan, Yemen, Bangladesh, Zimbabwe, Iraq, South Sudan, DRC and global FCAS. Reallocations occurred early in the pandemic, with respondents describing a lack of available funds for the initial COVID-19 response from emergency preparedness, which resulted in use of available funds from MNCHN. The primary use for the diverted funds reported was for IPC activities such as procurement of PPE, strengthening of water, sanitation and hygiene (WASH) services and to build additional inpatient capacity for isolation facilities. It was noted that even once additional funding did become available, it was frequently for COVID-19 specific measures only, and less so for maintaining essential services, including MNCHN services. In many cases additional human resources and supplies were also only available for COVID-19 related activities.

*“It took a while for the COVID funds to really come. So we had to use the existing funds to support the program during the adaptation and the later phases as well.”—Multilateral organisation, Bangladesh*

#### Planned funding and MNCHN activities which did not materialise

Six interviewees reporting on Yemen, Syria, South Sudan, Bangladesh, DRC, and global FCAS highlighted how the focus on COVID-19 meant that some other MNCH activities that were planned for 2020/2021 did not take place. These included trainings, quality improvement initiatives and entire MNCH programs in some cases. Examples cited included trainings, vaccination activities, HIV and TB, prevention of mother to child transmission (PMTCT), ANC and family planning (FP).

*“In many cases funds which had been expected before the pandemic for certain activities did not materialise and planned programs didn’t open. In Yemen we scaled back antenatal and delivery care in an area in the knowledge that another actor had received funding and would start delivering these services; however in the end this actor was not able to open the expected services, citing funding issues… Women were then not receiving these services and we were unable to meet the demand, frequently turning women away in labour. Home deliveries likely increased, and we saw increasing number[s] of neonatal tetanus, probably as a result”—NGO, Yemen*

### Disruptions to MNCH and nutrition services

Both the demand (service uptake) and supply (service provision) sides of essential MNCHN services in FCAS have been overwhelmingly affected by the pandemic. Factors affecting the demand side identified in literature and interviews included stigma, the fear of infection, interruption of service provision, real or perceived linkage of COVID-19 measures to punitive restrictions on movement and liberty (in particular for migrants and refugees), distance from health facility, difficulties with transportation, increased financial burden and fear of being quarantined following a positive test result [16, 24, 29, 94, 111, 115, 116, 118]. Factors affecting supply side according to literature and interviews included reduced staffing due to MNCHN staff being diverted to COVID-19, staff infected with or scared of contracting the virus at work leading to absences; stock outs and supply issues including PPE and routine medications; and overall deprioritisation of MNCHN [20, 56, 94, 116].

Within MNCHN services, interviews and literature also highlighted a reduction in the quality of care. For example, one online survey across 60 LMICs which includes some FCAS reports that due to understaffing, the rapid change of guidelines or unclear communication and challenges with the supply chain, the quality of maternal and newborn health services is perceived to be deteriorating [56]. Inadequate staffing levels and diversions of resources to COVID-19 were reported by interviewees as further factors contributing to reduced quality of care.

#### Sexual, reproductive, maternal and newborn health

Eight publications [39, 55, 62, 92, 94, 103, 115, 120], data from UNICEF’s dashboard, [16] and 21 interviews focused on Syria, Venezuela, Somalia, Afghanistan, Bangladesh, Zimbabwe, Iraq, Yemen, DRC, South Sudan, and global FCAS highlighted reduced and even complete suspension of the provision of sexual, reproductive, maternal and newborn services due to COVID-19. A key informant in an NGO reported that outpatient services were significantly reduced in many countries, often to half the normal activity. Interviewees highlighted that ANC services were reduced in many settings to reduce crowding and COVID-19 transmission and, in many cases, women were permitted only one ANC visit. PNC provision was reduced in Zimbabwe, Afghanistan, Venezuela, Bangladesh and South Sudan, with key informants in Bangladesh and Somalia reporting a complete suspension of newborn care [92]. Suspensions of outreach activities were common [39]. Interviewees described reduction in services that would normally have relied on outreach/mobile strategies as entry-points, including for reproductive health and sexual and gender-based violence (SGBV) interventions.

*“… In the camp, you have a really big component that's outreach activities. And this was seriously impacted by COVID-19. Outreach activities [were] almost zero for SRH, it almost disappeared completely… I didn't realize at first that it was the most important activity for SRH and SGBV.”—International NGO, Bangladesh*

Reduction in service uptake, including in ANC and facility delivery, was also reported across 13 publications [29, 56, 71, 86, 92–94, 106, 111, 113, 115, 118, 120], the data set [16] and 23 interviews focused on South Sudan, Nigeria, Bangladesh, Venezuela, Columbia, Afghanistan, Somalia, Zimbabwe, Iraq, Syria, Yemen, DRC and global FCAS. In Cox’s Bazar, ANC consultations for adolescents fell by 65% between January and May 2020 [106]. In Afghanistan, facility-based deliveries decreased by half [94]. Increased home deliveries with traditional birth attendants (TBAs) were reported in six publications, in several countries including Nigeria and Bangladesh [20, 29, 55, 92, 111, 116].

*“All other services including antenatal care were disrupted…in theory, the services were not stopped. But people stopped coming to the consultations. People stopped themselves obtaining services. And it was mainly because of the fear…They had the fear that maybe they go to COVID-19 from the hospital, from the healthcare providers…”—Multilateral organisation, Afghanistan*

*“The bigger concern was we had a huge reduction in the number of patients. So we went from… 2000 deliveries on average to, in July [2020], we had… 900 deliveries… I still don't know where all these people started to go.”—International NGO, Afghanistan*

According to key informants, unclear communication and lack of adaptation of COVID-19 messaging for pregnant women also led to confusion about going to health facilities to deliver or not as COVID-19 messaging was advising people to avoid attending them.

*“We did see a drop in service utilization. And I think that was probably for a number of reasons. One was….the lack of clarity in in terms of messaging, so should people stay at home or should people still come to the clinic.”—International NGO, South Sudan*

#### Child health

Downscaling and closure of consultations, reduced preventive and curative care, vaccination related to child health was seen in 13 publications [21, 22, 31, 32, 40, 44, 64, 69, 72, 73, 76, 80, 103], the Unicef dashboard [16] and 11 interviews focused on Venezuela, Syria, Afghanistan, Yemen, Bangladesh, Somalia, and multiple/global FCAS. Interviews and literature findings noted a decline in utilisation of outpatient and inpatient child health services [16, 24, 36, 73, 94]. Save the Children examined the impact of COVID-19 on child health in 37 countries and found that more than one third of respondents faced barriers to accessing healthcare including the closure of health facilities, long queues, and shortage of medications [105].

Routine immunisation was disrupted across many settings due to early recommendations from scientific advisory groups for emergencies to suspend all preventative mass vaccination campaigns, which placed children, especially those suffering from malnutrition, at increased risk [16, 32, 69, 73, 80, 103]. Additional reasons cited in the literature for vaccine disruption include delivery systems affected by the pandemic, healthcare services being stretched, social distancing measures, and caregivers becoming fearful of visiting health centres [28, 59, 94, 119]. Several interview respondents attributed the reduction or suspension in vaccination coverage to factors including supply chain issues, suspension of outreach activities to prevent COVID-19 spread and consequences of stopping other services (e.g., ANC) which normally would be used as an opportunity for comprehensive care provision, including vaccination.

The impact of suspensions in vaccination was highlighted in the literature [33, 51]. Despite the declaration of wild polio eradication in Africa in 2020, vaccine-derived polio transmission has increased and is now reported in a number of countries including Niger [32]. In Afghanistan, polio vaccination was stopped and there was a rise in polio cases, particularly in polio free areas [64, 72, 76]. Interview respondents also highlighted concerns of surges of new epidemics of childhood vaccine preventable diseases.

*“We are already seeing increases in vaccine-preventable diseases, for example Diphtheria, tetanus pertussis, and measles outbreaks. Polio campaigns were also suspended and Vaccine derived Polio cases are also occurring due to low vaccination coverage.”—NGO, setting anonymised on request*

Concerns have been raised over the effects of neglecting malaria control programmes including net distribution, seasonal malaria chemoprevention, and treatment, especially for children, since this age group has the highest burden of malaria, accounting for 70% of global malaria deaths [27, 48, 96, 121].

#### Nutrition

Nutrition services were suspended in several FCAS, including nutrition centres, food distributions and treatment for child wasting according to four publications [45, 78, 81, 94], the data set [16] and eight interviews focused on global FCAS, Bangladesh, Venezuela, Afghanistan, Syria and Somalia. Reasons for reduced nutrition activities cited in interviews and the literature included a link to reduction or suspension of community outreach activities, measures to prevent gatherings and prevent transmission of COVID-19, and diversion of nutrition staff to COVID-19 [73, 94].

*“We were told that you cannot go into the community because you can potentially take COVID in and out to the community… So we had to stop that…And because some of the programs, the nutritional programs such as dinners for kids were shut down… that is a problem because a lot of kids didn't have anything to eat except for what was given in those diners.”—NGO, Venezuela*

*“When the COVID-19 started… I would say nutrition really suffered, because… they are reassigning*… *all the nutrition staff to work on COVID-19 related activities. So, we noted that… all the upstream work that we do to do with policy guidelines… things were not moving, everything had to come to a standstill for like five months.”—Multilateral organisation, Somalia*

Reports from several countries refer to the difficulties that populations have been facing in accessing and ensuring food supplies. Reduced income, limited resources for quality diets, food insecurity, higher prices in food, limitation of health care services and interruption of humanitarian responses are leading to undernutrition [38, 45, 50, 83, 91, 105, 107, 109, 110, 114]. Interview respondents noted a reduction in uptake of nutrition services during the pandemic despite needs increasing in some contexts, especially for child nutrition interventions.

*“There was definitely a reduction in the number of children that were enrolled in supplementary feeding and therapeutic feeding programs. And this was… for a number of reasons. One of them being that you know, people were reluctant to come to the clinics.”—Multilateral organisation, Global*

#### Data collection activities

There was disruption or suspension in routine data collection, monitoring and evaluation and measurement activities in MNCHN according to 18 interview respondents reporting on Bangladesh, South Sudan, Somalia, Venezuela, Columbia, Afghanistan, Yemen, Syria and global FCAS. This was due to deprioritisation of MNCHN measurement activities, inadequate staffing, and difficulty in carrying out activities based in the community due to lack of PPE, movement restrictions and other COVID-19 prevention measures which leads to reduced quantity and quality of data. Multiple community measurement activities were suspended, including mortality surveys, [e.g., Standardised Monitoring and Assessment of Relief and Transitions (SMART) surveys] and nutrition surveys, as well as community surveillance, have also been impacted. As a result, there has been a significant reduction in the visibility of community level deaths of women and children during the pandemic.

*“I think everyone, even governments, are facing data collection gaps. Data collection and data quality was in a bad state before COVID-19 in many countries. And now with COVID-19, it's just worsening…we don't know how many people are affected. […] You know, in some countries, community health workers are very strong data collectors. But with COVID-19, they might not even go to the villages where they would normally go.”—Multilateral organisation, global*

### MNCH and nutrition outcomes

Findings from interviews and published literature point to increased morbidity and mortality associated with MNCHN across several settings related to the disruptions to funding and services, threatening years of advocacy and improvements [6, 43, 56].

#### Sexual, reproductive, maternal and newborn health outcomes

Increasingly late presentations which resulted in poorer health and mortality outcomes were reported in two publications [54, 111] and 11 interviews reporting on Venezuela, Bangladesh, Syria, Yemen, South Sudan and global FCAS. Women are reported as arriving late at heath facilities and more frequently presenting with serious complications (such as eclampsia) as ANC services are neglected. [54, 111] Interview respondents also reported women presenting with more severe maternal morbidity and complications linked to reduced pregnancy care and other COVID-19 related impacts, such as iron deficiency anaemia, secondary to increased levels of malnutrition.

*“I think one thing that we did see was an increase in late presentations and there was a subtle increase in maternal mortality cases in our clinic over the summer. That speaks to a presentation of patients not coming in for routine, preventative care in their pregnancy, and then having problems later on…”—International NGO, Bangladesh*

Estimations looking at the indirect mortality from COVID-19 disruptions in LMICs, indicate that over six months of disruptions would result in an increase of 8.3%-38.6% in maternal deaths per month across 118 countries [26]. Another publication estimated that, over a year in LMICs, a 10% decline in coverage of maternal and newborn health services would mean an additional 1.7 million women who give birth and almost 2.6 million newborns that would experience major complications without receiving the needed obstetric and newborn care. This would result in additional 28,000 maternal deaths and 168,000 newborn deaths [63]. Data from the four most populous LMICs (including Nigeria) could see a 31% increase in maternal and newborn deaths and still births as a result of reduced FP, ANC and facility based deliveries in the next 12 months [100, 111]. Authors caution that these numbers are an underestimation, especially in rural, low resource and FCAS where the most vulnerable pregnant women are [61]. Specific estimates for selected FCAS indicate that the impact of a large service disruption in 2020–2021 would lead to a 1–11% increase in maternal mortality; and that between 10,700 and 725,900 women would be left without access to facility-based deliveries across several FCAS, such as in Yemen, South Sudan and Central African Republic (CAR) [97–99, 101, 102, 104, 112].

Nine interview respondents reporting on Bangladesh, Yemen, Syria and global FCAS also highlighted an increase in maternal mortality across a range of humanitarian settings, citing inability to access care, reduced care-seeking behaviour, reduced ANC and potential COVID-19 infection itself as attributing factors.

*“…during my first three months of being in Yemen, I had no maternal deaths… then when COVID hit, we had so many more maternal deaths. Sometimes, it's hard for us to say exactly whether it was because of COVID-19 because we didn't actually test everybody. […] It could have been due to COVID. It could have been that people were a lot more afraid of coming to the hospitals, because obviously that was like, I think, a common narrative around the world.”—International NGO, Yemen*

#### Child health

The impact on child health was highlighted by 19 publications [26, 33, 35, 37, 46, 73, 74, 79, 89, 92, 94, 97–99, 101, 102, 104, 112, 116] and eight interviews focused on Bangladesh, Venezuela, Yemen, Syria and global FCAS. A report from Save the Children warned that 60 million children will need humanitarian assistance in 2021 to survive, which accounts for half of all children in need globally [74]. A modelling study that considers the excess mortality due to disruptions in essential maternal and child services and access to food reported estimated 253,500 to 1,157,000 additional child deaths in a year in the least and most severe scenarios respectively [26]. This translates to a 9.8–44.7% increase in deaths in under-fives per month across 118 countries LMICs and to a 12–18% increase in selected FCAS [26, 97–99, 101, 102, 104, 112].

Interview respondents highlighted increased child morbidity and mortality with children frequently arriving at health facilities late and with severe disease. Key attributing factors included reduction in child health provision facilities, reduced outreach activities, reduced health seeking behaviour and worsening malnutrition.

*“…In Yemen, there were a lot of children arriving in a very bad state. In White Nile the team asked because the number in the hospital was very low. And that was because the activities—the screening activities at community level stopped with the pandemic.”—International NGO, Yemen and Sudan*

There is a strong argument to continue routine vaccinations, ensuring appropriate use of PPE, hygiene and physical distancing measures are put in place [46]. A modelling study estimated that routine childhood immunisations in selected African countries should be continued as the deaths prevented by this activity outweigh the risk of COVID-19 deaths related with the visits to vaccination clinics [35]. Catch-up programmes in some FCAS were restarted to address the impact of early programme disruptions [79, 92].

#### Nutrition

Increased risk of malnutrition and worsening nutrition outcomes were reported in 17publications [26, 60, 73, 77, 79, 81, 83, 84, 87, 88, 90, 92, 94, 109, 110, 116, 118] and nine interviews focused on global FCAS, Syria, Venezuela, DRC, Yemen, and Bangladesh. Several settings reported rises in severe acute malnutrition admissions, ranging from increases of 10% up to 70% [73, 77, 87, 94, 109, 118]. Several interviews reported increased malnutrition, with women and children being most at risk, particularly in settings that were already experiencing high levels of food insecurity and malnutrition before COVID-19. Women’s nutrition was also affected with at least a quarter of a million pregnant and lactating women, in Southern Yemen, in need of malnutrition treatment [87, 90]. In a modelling study looking at the indirect mortality from COVID-19 in LMICs including FCAS, the authors estimate that 18–23% of additional deaths in children under-five will be caused by the increased prevalence of wasting [26].

### Adaptation strategies for MNCH and nutrition services

The literature search and key informant interviews reported a range of adaptation strategies for MNCHN implemented, most of them with the primary objective of reducing transmission of COVID-19.

Factors enabling successful adaptations of MNCHN services included preparedness, coordination and collaboration between actors, good communication, provision of adequate amount of supplies and support from the local population [75, 81].

Barriers to adaptations include lack of flexible funding, challenges with global supply chains, and capacity to rapidly implement new ways of working [78]. There were concerns from some key informants about whether the priority placed on reducing transmission of COVID-19 compared to other essential services was proportionate to the threat given the seriousness of other factors affecting MNCHN in FCAS. Acceptability of adaptations by the local population was a further challenge faced by several actors. For example, lack of acceptance of one adaptation preventing hospital visitors led to reduced service uptake.

*“…The first thing we did was to stop with all the caretakers to enter the hospital, you need to think that in that area, I mean the patient, they are always coming with the mother-in-law… And this was a big, huge thing. And challenge, because also, we need to have a lot of meetings with the elderly, with the community to also make them understand, we need to reduce the flow of the people coming inside and going outside.”—International NGO, Afghanistan*

*“People started losing trust in our facilities because they came and they didn’t get treated, because there wasn’t anything, which is very unusual… I think that had a very, very huge impact on the perception of the community of what we do.”—International NGO, DRC*

#### Adaptations to funding

There were some accounts of positive adaptations in relation to funding MNCHN services in FCAS. One study highlighted that the pandemic has diversified the funding sources which could be a solution to strengthen reproductive, maternal and child health programmes [117]. 11 interview respondents reporting on South Sudan, Bangladesh, Syria, Afghanistan, Venezuela, Somalia, Yemen and global FCAS reported additional funding being made available from both international internal organisation funding and from donors. Strengthening WASH including IPC was a major reported use for the funding to increase availability and quality of WASH in health facilities and improve communication and education on WASH which had a positive effect beyond the avoidance of COVID-19 infections. Various actors and funders highlighted that funding was not earmarked for the pandemic and could be used for other activities such as MNCHN services.

*“On the one hand… IPC improved, hygiene improved, hand washing improved, I mean, it was much more focus put on that, where you could actually even see a reduction in certain diseases…diarrhoeal diseases, and whatever.”—International NGO, DRC*

#### Infection prevention and control measures

Reorganisation of patient flow, social distancing measures (e.g., reduction of hospital visitors, reduction in access to smaller groups of patients at a time, reduced frequency of facility visits), reduced opening hours and reduction or suspension of out-patients department (OPD) consultations were highlighted in six publications and 16 interviews [20, 34, 55, 86, 113, 117] and 16 interviews focused on Syria, South Sudan, Somalia, Afghanistan, Nigeria, Venezuela, Yemen, Bangladesh, Iraq and global FCAS.

*“These are services that are usually very crowded. So they tried to adjust the services too. They had longer hours. They gave women specific times to come to reduce some crowding, where they did have to decrease services, they prioritised women that were in the third trimester of pregnancy.”—Multilateral organisation, global*

Interview respondents noted that increased WASH activities were implemented in many contexts, with handwashing and environmental IPC prioritised. Emphasis was placed on training staff in IPC and use of PPE in several settings [68].

#### Use of technology

Telemedicine was used as an adaptation strategy in a number of FCAS according to four publications [34, 56, 86, 113] and eight interviews focused on Venezuela, Columbia, Afghanistan, Yemen, Bangladesh and global FCAS. Several interview respondents highlighted that MNCHN service provision was shifted to online modalities, for example mobile phone/WhatsApp calls and messaging. In addition to its use for providing services and information to users, interview respondents noted the use of online technology for staff training (including on COVID-19 measures and for maintaining essential health services) and to adapt data collection in several settings to try and continue the delivery of routine monitoring and evaluation on MNCHN data.

*“They can do the consultation remotely, using the tablet, community health workers, you know, bring it to the pregnant woman and then they consult with the midwives. And then for the other thing also that we also try to improve in is using the mobile phone platform […]—the community can text a message to the health provider or to the community health workers about their problems.”—Governmental agency, Bangladesh*

Nevertheless, the impact of limited use of telemedicine services has not been equitable across settings and socioeconomic groups. Some respondents noted that access and proficiency in technology was a challenge for adaptation strategies involving online tools for staff and target populations; and literature highlighted that it might not be a realistic solution for some settings [30].

#### Decentralisation of services

Due to the decrease in demand for facility-based health care, it was crucial to provide MNCHN services at the community level according to two publications and 13 interviews [56, 117] and 13 interviews focused on Bangladesh, Venezuela, Yemen, Somalia, South Sudan, Afghanistan, Zimbabwe, Syria, Venezuela and global FCAS. Several interview respondents intended to shift MNCHN services to mobile or outreach services to better reach populations in the community. Examples of how they managed to operationalise this shift included using important community gathering places such as the local mosque to provide health promotion and related services.

*“We decided to use a mobile clinic to do outpatient treatment… We just needed to be closer to the population to do this screening and treatment at the community level. We left the hospital and moved to the community to try to treat the malnourished children earlier.”—International NGO, Yemen*

Interview respondents described new communication and advocacy activities on maintaining essential services to reassure and continue promotion of usage of MNCHN services, such as institutional deliveries.

*“Community messaging has been a key part of what we’ve done. To reassure people that the services are still open and safe to attend. And also information about COVID, how to stay safe, hand washing, guidance, all those kinds of things that we’ve seen in many other contexts globally.”—International NGO, Yemen*

Another form of decentralisation of services was using self-care interventions. Several respondents mentioned the shift in global nutrition guidance to using mother/caregiver led mid upper arm circumference (MUAC) measurements to screen for malnutrition at home, with guidance on when to seek health care given to caregivers. This adaptation was also highlighted in the literature [16, 75, 81].

Interview respondents also described how treatment protocols in some settings were altered to continue essential healthcare whilst reducing contact frequency at facilities. Measures included giving longer durations of medication and food rations and encouraging use of long-acting reversible contraceptives (e.g., intrauterine devices, implants).

However, some interview respondents noted that despite the intention to increase community-based services, the extent to which this has actual been possible has been limited in many settings. This was attributed to funding constraints, the repurposing of community health workers to COVID-19 related activities, and limitations to community gatherings. In some countries, outreach activities were suspended during the pandemic.

## Discussion

Our findings demonstrate that in many cases funding for MNCH and nutrition in FCAS has been diverted, reduced, or suspended during the COVID-19 pandemic. Supply and use of MNCHN services has also been disrupted due to a combination of factors, with reported increased morbidity and mortality across MNCHN. Humanitarian actors have adapted and implemented strategies to improve access to MNCHN interventions as part of the pandemic response, but in most FCAS included in the study, they have been limited, insufficient to account for overall disruption to services, and subject to inequities in their provision. Across the study, the findings from our literature search and interviews were generally complementary.

This study highlights the impacts of the COVID-19 pandemic on funding of MNCHN, and particularly the deprioritisation of funding by governments in favour of resource allocation to COVID-19 specific measures. Diversion of funding away from MNCH has previously been documented across prior infectious disease outbreaks and epidemics including Ebola and Zika [10]. There is a clear call for governments to firstly ensure that funding for MNCHN is re-established to pre-pandemic levels. Given the reported impacts on reduced service provision and uptake and resulting poor MNCHN outcomes, additional funding should be leveraged to address the collateral impacts on MNCHN. A greater focus should be placed funding for health system strengthening activities. Emergency preparedness plans must include capacity and funding to maintain MNCHN activities.

Supply- and demand-side challenges to MNCHN services have also been seen across previous large-scale infectious disease outbreaks, particularly in contexts with fragile health systems [124]. Our study findings suggest the integration of the COVID-19 response into essential health care as a possible way to develop long term strengthening of fragile health systems [41].

Although limited and incomplete, available evidence shows that preventable deaths are occurring due to COVID-19 disruptions in MNCHN services. Evidence from previous pandemics shows that indirect impacts can be greater than the disease itself [124]. Whilst empirical research reporting on wider health implications of COVID-19 in humanitarian settings remains scarce, a series of case studies on the collateral impact of COVID-19 on non-communicable diseases across six humanitarian settings highlights reduced access to and provision of health services as well as financial and human resource diversion towards COVID-19—similar to the findings of our study [5].

Literature drawing on lessons from previous outbreaks highlights the importance of maintaining essential general health services in humanitarian settings, ensuring access to IPC/WASH for displaced populations and promoting community trust and engagement as part of effective public health measures [7]. In addition to ensuring prioritisation of MNCHN and maintenance of essential services in times of additional crisis, a holistic public health approach should be taken to reduce supply and demand barriers. Surge and emergency preparedness planning is crucial to prevent stock outs and supply issues, and protection of healthcare and other front-facing staff is a priority to continue services. Community engagement, rumour management and clear communication are all important to understand and alleviate concerns. Opportunities to combine community preventive activities together should be explored to increase efficiency and expedite catch-up activities such as nutritional interventions, mass vaccination campaigns, Vitamin A, bed nets, seasonal malaria chemoprophylaxis (SMC), and other activities.

Whilst limited in some settings, our finding of a range of adaptation strategies for MNCHN is echoed across other studies [10, 20]. The importance for documenting and sharing these solutions has been highlighted [125]. Additionally, capacity building and continued training of staff is critical to implementing adaptations. Integration of monitoring and evaluation into adaptations with regular reviews is also vital to measure impacts. Some studies included from the literature review also note that investing in primary health care and in the integration of COVID-19 response into essential health care could be the way to strengthen already fragile health systems [41, 43]. Donors should make available additional funding to enable adaptations to be fully implemented.

To our knowledge, this is the first study to investigate the collateral impact of COVID-19 on MNCHN across multiple FCAS. Our study is reasonably extensive in that it includes data from the literature and interviews from the majority of FCAS (51.28%). Interviews covered a range of actors including funders, staff at the global level in multilateral organisations, humanitarian field staff and clinical staff. We aimed to ensure that this study was comprehensive, utilising snowballing to identify further interview participants and utilised data provided by the key informants. The triangulation of the data allowed for a broader analysis of the current situation, strengthening our findings.

However, this study also has several limitations. Through the range of settings in which data collection was undertaken, this study emphasises breadth in its findings and recommendations. Context matters, and therefore the findings and recommendations will not be generalisable to every FCAS. Future research should be designed to analyse regional or other contextual trends. Although we included English, Portuguese, Spanish, and French articles in the review, we may have missed relevant articles published in other languages. All included peer-reviewed articles were observational studies, and thus causation cannot be attributed. Whilst the use of online modalities for interviews undoubtedly increased the ability for data collection, a documented challenge of research within FCAS is poor internet access [126]. It is therefore possible that information from additional key informants was missed if they were unable to participate due to internet access issues. Crucially, this study does not include the voices of individuals from affected communities. Some humanitarian data sources were also not able to be included due to the design, for example food security data. It was not feasible to collect data from populations directly affected within the bounds of this study, but as a result these findings and recommendations should be taken with some caution as they may not be representative of the views of patients and service users in affected populations. Lastly, it is important to consider the positionality of the researchers and the associated political, social, economic and historical contexts and power hierarchies that occur when doing research in FCAS [127]. This study was led by a team of high-income country researchers, none of whom are from affected communities within FCAS. This will have impacted the study design, data collection and interpretation; and findings should again be considered in light of this.

We do not yet know the full extent of the impact of COVID-19 related disruptions on MNCHN outcomes. Since existing data collection, including mortality surveillance and surveys, and data analysis have been disrupted, limited evidence exists evaluating the impacts of demand and supply disruptions across the settings. Further work should seek to further gather rigorous data and evaluate the impacts of COVID-19 on MNCHN in FCAS so that future programmes can be best adapted to prioritise, meeting the needs of women and children.

## Conclusions

Our study findings show that the COVID-19 pandemic is exacerbating negative MNCHN outcomes in FCAS that were already significantly worse compared to other settings before COVID-19 due to lack of resources and the deprioritisation of essential services for women and children. These findings also demonstrate that lessons learned from previous epidemics on reducing their indirect impacts on MNCHN have not been systematically integrated into humanitarian responses in FCAS during COVID-19. While humanitarian actors have made several adaptations to continue providing MNCHN services during the pandemic, these strategies have often been implemented unevenly within and across settings, and not been evaluated.

Political will and mobilising of sufficient resources, with sustained commitment from donors, governments, NGOs and multilateral organisations working in FCAS are urgently needed to prevent further negative impacts on mothers and children. Steps must be taken to strengthen health systems and existing MNCHN services, ensuring that the most vulnerable populations are reached. Timely data collection is also needed to be able to use data to inform MNCHN programming in real-time, and research should be embedded within MNCHN programming in FCAS to assess the effectives of implementation strategies. Continued advocacy efforts are also vital to the prioritisation of MNCHN services, with particular attention needed to mitigate the predicted negative secondary effects of COVID-19 on MNCHN outcomes in FCAS over the coming years.

## Supplementary Information

**Additional file 1.** Search terms for scoping review.

**Additional file 2.** List of included countries as per World Bank.

**Additional file 3.** Interview Guide.

**Additional file 4.** Characteristics and key findings of literature included in the review.

## Data Availability

The datasets generated and analysed during the current study are not publicly available due to the protection of individual privacy.

## Abbreviations

ANC: Antenatal care
CAR: Central African Republic
COVID-19: Coronavirus disease 2019
DRC: Democratic Republic of Congo
FCAS: Fragile and conflict-affected settings
FP: Family planning
IPC: Infection prevention and control
IRC: The International Rescue Committee
LMIC: Low- and middle-income country
MNCH: Maternal, newborn, and child health
MNCHN: Maternal, newborn, child health and nutrition
MSF: Médecins Sans Frontières
MUAC: Mid upper arm circumference
NGO: Non-governmental Organisation
PMTCT: Prevention of mother to child transmission
PNC: Postnatal care
PPE: Personal protective equipment
OPD: Out-patients department
SGBV: Sexual and gender-based violence
SMART: Standardised monitoring and assessment of relief and transitions
SMC: Seasonal malaria chemoprophylaxis
SRH: Sexual and reproductive health
TBA: Traditional birth attendant
UN: United Nations
UNFPA: United Nations Population Fund
UNICEF: United Nations Children's Fund
WASH: Water, sanitation and hygiene
