# Supplementary material for: A mixed methods study to assess the impact of COVID-19 on maternal, newborn, child health and nutrition in fragile and conflict-affected settings
Source: Confl Health. 2022 Jun 3;16:30. doi: 10.1186/s13031-022-00465-x (PMC9162897; doi:10.1186/s13031-022-00465-x)
Supplement: Supplementary file 1 — Additional file 1. Search terms for scoping review. [file 13031_2022_465_MOESM1_ESM.docx]

**Additional file 1: Search terms for scoping review**

Search algorithm: (Population AND health services AND settings AND COVID-19)

1) POPULATION

| **Broad Topic** | **Search terms** |
| --- | --- |
| Maternal | Maternal OR antenatal OR ante-natal OR prenatal OR pre-natal OR gestati* OR childbirth OR birth OR intrapartum OR obstetric* OR labo?r OR pregnancy OR vertical OR postpartum OR post-partum OR puerper* OR perinatal OR maternity OR reproductive OR birth attendant OR skilled birth OR c?esarean OR c?section OR eclampsia OR pre-eclampsia OR preeclampsia OR fertil* OR f?tal OR |
| Newborn | Newborn OR new-born OR new born OR postnatal OR post-natal OR neonat* OR kangaroo OR low birthweight OR low birth-weight OR pre?term OR prematur* OR post?term OR skin-to-skin OR breastfeeding OR breast-feeding OR lactat* OR formula-feeding OR bottle-feeding OR breastmilk substitute OR |
| Child | Child* OR childhood OR infant OR postneonatal OR under five OR under-five OR underfive OR under 5 OR p?ediatric* OR growth OR infant feeding OR infant nutrition OR |
| Adolescent | Adolescent* OR teen* OR youth* OR young adult* OR school-age OR young mother* OR young person* OR young people OR young wom?n OR |
| Combination of groups | Vulnerable population* |

2) HEALTH SERVICES

| **Broad Topic** | **Search terms** |
| --- | --- |
| Health care | (Health adj3 delivery) OR (health adj3 utili?ation) OR (health adj3 services) OR (care adj3 provision) OR health-care OR healthcare OR (care adj3 worker*) OR (community adj3 worker*) OR obstetrician* OR midwife OR midwives OR nurse* OR (medical adj2 officer*) OR (clinical adj2 officer*) OR |
| Health system | Health system* OR health facilit* OR maternity OR referral OR facility-based OR home-based OR school-based OR community-based OR |
| Provision/use | Provision OR coverage OR essential services OR essential healthcare OR indirect effect* OR collateral damage OR maintain* OR resilien* OR strengthen* OR sustain* OR mitigation strateg* OR cash transfer* OR user fee* OR helpline* OR digital OR telemedicine OR tele-health OR routine service* OR ICCM OR BEmONC OR CEmONC OR nutrition OR vaccination* OR immuni?ation* OR malaria OR outbreak* |

3) SETTING

| **Broad topic** | **Search terms** |
| --- | --- |
| FCAS | (Fragile adj2 (state* or setting*)) OR (disaster adj3 (relief or plan*)) OR ((relief or aid or rescue) adj2 work*) OR ((armed or zone) adj2 conflict*) OR (conflict affected adj3 (population* or person* or communit*)) OR conflict setting* OR war* OR (refugee* or evacuee or evacuated) OR ((force* or population or human or internal*) adj2 displace*) OR (internally displaced adj2 (person or people)) OR humanitarian OR |
| FCAS Countries (as per World Bank ) | Afghanistan OR Libya OR Somalia OR Syria OR Bangladesh OR Rohingya OR Burkina Faso OR Cameroon OR Central African Republic OR Chad OR Democratic Republic Congo OR Iraq OR Mali OR Mozambique or Myanmar OR Niger OR Nigeria OR South Sudan OR Yemen OR Burundi OR Republic Congo OR Eritrea OR Gambia OR Guinea-Bissau OR Haiti OR Kosovo OR Lao OR Lebanon OR Liberia OR Papua New Guinea OR Sudan OR Venezuela OR West Bank OR Gaza OR Zimbabwe OR Comoros OR Kiribati OR Marshall Islands OR Micronesia OR Solomon Islands OR Timor-Leste OR Tuvalu |

4) COVID-19

**Database search restrictions:**

- Restrict to “Human” when possible (in Medline and Embase)
- Limit to dc=20200301-20210131 [March 1st, 2020 to January 31th, 2021] (In Embase and Medline)
- Limit to2020 – 2021 (in Global health)
