# Supplementary material for: A mixed methods study to assess the impact of COVID-19 on maternal, newborn, child health and nutrition in fragile and conflict-affected settings
Source: Confl Health. 2022 Jun 3;16:30. doi: 10.1186/s13031-022-00465-x (PMC9162897; doi:10.1186/s13031-022-00465-x)
Supplement: Supplementary file 2 — Additional file 2. List of included countries as per World Bank. [file 13031_2022_465_MOESM2_ESM.docx]

**Additional file 2: List of included countries as per World Bank**(1)

Afghanistan

Libya

Somalia

Syria Arab Republic

Burkina Faso

Cameroon

Central African Republic

Chad

Democratic Republic of Congo

Iraq

Mali

Mozambique

Myanmar

Níger

Nigeria

South Sudan

Yemen

Burundi

Republic of Congo

Eritrea

The Gambia

Guinea-Bissau

Haiti

Kosovo

Lao People Democratic Republic

Lebanon

Liberia

Papua New Guinea

Sudan

Venezuela

West Bank and Gaza (territory)

Zimbabwe

Comoros

Kiribati

Marshall Islands

Micronesia

Solomon Islands

Timor-Leste

Tuvalu

1. The World Bank. FY21 List of Fragile and Conflict-affected Situations. FCS List. 2020.
