# Supplementary material for: A mixed methods study to assess the impact of COVID-19 on maternal, newborn, child health and nutrition in fragile and conflict-affected settings
Source: Confl Health. 2022 Jun 3;16:30. doi: 10.1186/s13031-022-00465-x (PMC9162897; doi:10.1186/s13031-022-00465-x)
Supplement: Supplementary file 3 — Additional file 3. Interview Guide. [file 13031_2022_465_MOESM3_ESM.docx]

**Appendix 3: Interview Guide**

**Impact of COVID-19 on maternal, newborn, child health and nutrition in fragile and conflict affected states**

| **Interview Details** *(complete prior to the interview)* | |
| --- | --- |
| Date of the interview |  |
| Name of the interviewer |  |
| Email of interviewer |  |

*(Please briefly explain the following to the respondent before starting)*

**Objective**

Our objective is to gather evidence on the impact of COVID-19 on maternal, newborn and child health (MNCH) and nutrition in fragile and conflict affected states (FCAS) and to capture examples of how humanitarian organizations are adapting their existing programs or innovating new programs to address COVID-19 in their unique environments. These interviews aim to understand facilitators and barriers to programme implementation and identify adaptation and innovative examples among humanitarian and development partners engaged in MNCH and nutrition in FCAS during COVID-19.

This is to support UNICEF UK in advocating to the UK Government to continue to play a leading role in supporting MNCH and nutrition programming in FCAS and to ensure the global COVID-19 response does not divert or de-prioritise critical funding away from lifesaving and essential health services for women and children.

Our research intends to broadly address the following questions:

- **What** is the impact of COVID-19 on MNCH and nutrition outcomes in FCAS?
- **Which** examples/interventions/best practices can be helpful to inform UK Government’s approach and work on ending preventable maternal, newborn and child morbidity and mortality in FCAS?

This data will also to contribute for the [COVID-19 Humanitarian Platform](https://www.covid19humanitarian.com/). This platform was created to gather, curate, analyze, interpret and disseminate COVID-19-specific and -sensitive interventions that are being implemented in a variety of humanitarian settings. The goal is to facilitate the sharing of context-specific field experiences about how humanitarian programs are responding to and being adapted to the COVID-19 pandemic. The website will host both technical guidelines as well as operational field experiences from humanitarian actors in different settings.

This set of interviews will fall into the category of COVID-19-sensitive interventions (adaptations), nevertheless ensure you are familiar with the operational framework before starting the interview.

**Data privacy and consent:**

The interview will take approximately 30 minutes. We ask for your name and email in order to contact you for possible clarifications. However, your submission will be de-identified to remove any personal information. We now ask for your oral consent to *(check boxes)*:

|  | Record the interview |
| --- | --- |
|  | Display the name of organization and location |
|  | Upload the information to the COVID-19 website |
|  | Use the information for other analysis |

| **A. Respondent details** *(optional, depending on consent answer. In all cases these data are for follow up purposes only, will be stored securely and not be published)* | |
| --- | --- |
| **Name** |  |
| **Email** |  |
| **Position** |  |
| **Notes** |  |

| **B. Intervention Details** *(these relate to the setting of the ONE intervention specified in section I)* | |
| --- | --- |
| **Organisation name** |  |
| **Country of operations** |  |
| **Specific Location** *(e.g. region, town)* |  |
| **Urban/Rural** | \|  \| Urban \| \| \| --- \| --- \| --- \| \|  \| Rural \| \| \|  \| Mixed \| \| \|  \| Other *(please specify)* \|  \| |
| **Country Income Level** | \|  \| LIC \| \| --- \| --- \| \|  \| MIC \| \|  \| Other *(please specify)* \| |
| **Population status** | \|  \| Refugee \| \| --- \| --- \| \|  \| IDP (Internally Displaced People) \| \|  \| Asylum seeker \| \|  \| Non-displaced \| \|  \| Migrant \| \|  \| Mixed \| \|  \| Other *(please specify)* \| |
| **Setting** | \|  \| Camp/settlement \| \| --- \| --- \| \|  \| Non-camp/settlement \| \|  \| Mixed \| \|  \| Other *(please specify)* \| |
| **Date of first COVID-19 case** |  |

| *(Return to these checkboxes after learning about the intervention below)* | |
| --- | --- |
| **Framework categorisation** *(This relates to the* ONE intervention specified in section I) | |
| **Framework category**  *(select one)* | \|  \| Preparing for and Responding with COVID-19 specific interventions \| \| --- \| --- \| \|  \| Adapting existing interventions to COVID-19 \| \|  \| Cross cutting issues \| |
| **Framework section**  *(select one)* | \|  \| Risk assessment and context analysis \| \| --- \| --- \| \|  \| Investigation & surveillance \| \|  \| Prevention \| \|  \| Case management \| \|  \| Prioritization \| \|  \| Human Resources \| \|  \| Essential Health Services \| \|  \| Other programmatic areas \| \|  \| Coordination \| \|  \| Ethics \| |
| **Framework area**  *(If multiple, please note the* ***most relevant*** *framework area in the notes)* | \|  \| Health facility assessment \| \| --- \| --- \| \|  \| Context analysis \| \|  \| Investigation \| \|  \| Surveillance \| \|  \| Isolation measures \| \|  \| Rumour management \| \|  \| Infection Prevention and Control \| \|  \| Risk Communication and Community Engagement \| \|  \| Triage \| \|  \| Management of mild cases \| \|  \| Management of severe cases (non-ICU) \| \|  \| Management of severe cases (ICU) \| \|  \| Maternal Child Health \| \|  \| Sexual Reproductive Health \| \|  \| Mental Health \| \|  \| Communicable diseases \| \|  \| Non-Communicable Diseases \| \|  \| Injury and Rehabilitation \| \|  \| Nutrition \| \|  \| Food Security \| \|  \| WASH \| \|  \| Education \| \|  \| General protection \| \|  \| Sexual and Gender- Based Violence \| \|  \| Diversity and Inclusion \| |

| **C. Organisation’s Activities** |
| --- |
| 1. Could you please briefly describe the organisation’s areas of activity in this setting?  Prompts:   - How long have they been in the country, which were the normal areas of activity? |
|  |
| **D. Funding** |
| Was any funding for MNCH or nutrition activities diverted to COVID-19? Please explain.  Prompts:  If so, which donors diverted their funds?  Does your organisation usually receive any funding from UK Aid? If so, did this support change with the pandemic? |
|  |
| 1. What would be your recommendation on priority funding gaps in MNCH or nutrition in your setting?(specifically, funding gaps exacerbated by COVID-19)   Prompts:   - Please specify the services which were maintained and which were de-prioritised (eg. maternal health was safeguarded but vaccination was not) |
|  |
| 1. What would help mitigate these funding gaps if second wave restrictions come into place or in face of future crisis?   Prompts:   - For e.g. the need for longer term flexible funding arrangements to allow for adaptation. |
|  |
| **E. Other Actors** |
| 1. What level of priority has there been for MNCH and nutrition during the pandemic? From governments, donors, NGOs and communities.   Prompts:   - E.g. Pressure from governments for COVID-19 treatment centres, or to convert MNCH facilities to treat COVID-19 patients, diversion of IPC supply from these services. |
|  |
| How was the capacity of other actors in the region affected by COVID-19?  Prompts:  Did your organisation witness any impact from downscaling/closure of services by other actors? |
|  |
| **F. MNCH or nutrition service utilisation and outcomes** |
| 1. Was the activities coverage affected? Did you see a drop in pregnant women and children using services? If so, in which ones? |
|  |
| 1. Can you please tell me about COVID-19’s indirect impact on MNCH and nutritional status of communities and patients? For example, do you have any qualitative or quantitative data illustrating the mentioned COVID-19’s impact on MNCH service utilisation and/or MNCH outcomes? |
|  |
| **G. Measurement Activities** |
| How was the epidemiological information about MNCH or nutrition affected during COVID-19 pandemic? |
|  |
| Were measurement activities (e.g. mortality surveys, nutrition surveys, routine data collection including M&E etc.) suspended or delayed due to COVID-19? If yes, please explain. |
|  |
| **H. WASH Interventions** |
| 1. What was the impact of COVID-19 on the WASH interventions in the MNCH or nutrition activities?   Prompts:   - Was there a decrease/increase in the quality of these activities? - Was it possible to continue to implement IPC measures across the MNCH and nutrition activities as usual? |
|  |
| **I. Maternal, Newborn, Child Health and Nutrition Activities** |
| 1. Could you describe what changes happened in your MNCH or nutrition activities since the start of the COVID-19 pandemic?  Prompts:   - Did the activities stop? If so, which ones, when and why they were stopped? (e.g. funding diverted to COVID-19, change of prioritisation focus) - Were there any planned activities that did not go ahead? Which ones and why? - Did they continue? If so, which ones? |
| *(Please mark the activities that were stopped and briefly mention when and why were they stopped)*   \| **Activities** \| \| **Brief explanation of when and why activities stopped** \| \| --- \| --- \| --- \| \|  \| Antenatal Care \|  \| \|  \| Deliveries \|  \| \|  \| Postnatal Care \|  \| \|  \| Newborn Care \|  \| \|  \| Vaccination \|  \| \|  \| Child health consultation \|  \| \|  \| Nutrition centre \|  \| \|  \| Bed nets distributions \|  \| \|  \| Food distributions \|  \| \|  \| Others: \|  \| |
|  |
| 2. If the activities continued, did they continue as usual? Were they adapted? Did you add a new activity in your program, or change its focus or the way/modality it was implemented? If so, choose ONE intervention and briefly explain the intervention and the rationale for the adaptation/innovation (how and why)?  Prompts:   - Main intervention activities and modalities - Specific steps taken - Timing between first COVID case/start of the crisis in that context - Basis/guidance upon which the response/adaptation was made |
|  |
| 1. Why did you decide to adapt it this way? Explain the contextual considerations amongst other issues.   Prompts:   - Community dynamics and community perception (of the organisation/humanitarian actors; of COVID-19) - Social, cultural and/or religious factors - Relations between organisation, community groups, government, other partners - Existing systems (health system, food systems – e.g. markets, etc…), structures, facilities (health, WASH etc…), logistics - Governance: government/non-government control of the area, national protocols/plans - Humanitarian access and security issues - COVID-19 transmission scenario at the time of design and projections |
|  |
| 1. What challenges did you have in this adaptation? Which factors enabled your adaptation to be implemented? Which were most important challenges/enabling factors?   Prompts:   - Contextual factors (e.g. bad relationship with health district, lack of coordination among humanitarian actors, position of community leaders) - Internal organisational factors (e.g. human resources gaps, supply stock, presence of an emergency team with expertise in outbreaks, focus on this area by the organisation) |
|  |
| 1. Which resources (financial, human, supplies/logistics) did you need for this adaptation?   Prompts:   - ‘Hardware’ inputs needed e.g. human resources, supplies, funding - ‘Software’ inputs needed e.g. strong leadership, commitment, community support, political buy-in |
|  |
| 1. Is it working as intended or not? Please explain regardless of outcome.   Prompts:   - Self-evaluation of organisation - Community reactions/perceptions - Feedback from beneficiaries |
|  |
| **J. Other** |
| 1. Any other comments/information that you would like to share?   Prompts:   - Additional documents/reports/tools - Recommendation of other colleagues/organisations to contact - Feedback on the COVID-19 Humanitarian Platform (how could it be more useful?) - Ask them to please circulate online share experience form to their partners |
| *(Please add any links or note file locations. Note any restrictions on sharing)* |
