# Supplementary material for: A mixed methods study to assess the impact of COVID-19 on maternal, newborn, child health and nutrition in fragile and conflict-affected settings
Source: Confl Health. 2022 Jun 3;16:30. doi: 10.1186/s13031-022-00465-x (PMC9162897; doi:10.1186/s13031-022-00465-x)
Supplement: Supplementary file 4 — Additional file 4. Characteristics and key findings of literature included in the review. [file 13031_2022_465_MOESM4_ESM.docx]

**Appendix 4: Literature review findings**

Table 1. Characteristics and key findings of peer-reviewed publications

| **Author and year** | **Study design** | **Setting** | **Target population** | **General topic** | **Key findings** |
| --- | --- | --- | --- | --- | --- |
| Abbas K, Procter SR, van Zandvoort K et al. (2020)(1) | Modelling | Africa | Children | Child health | Authors estimated the deaths prevented by continuing routine vaccination activities outweigh the excess risk of COVID-19 deaths related with the implementation of these activities. |
| Abdulah DM, Abdulla BMO, Liamputtong P. (2020)(2) | Qualitative | Iraq | Children | Child health | Children (6-13 years old) experienced stress, loneliness and sadness due to the confinement and the possibility of COVID-19 infections. |
| Ahmed SAKS, Ajisola M, Azeem K, et al. (2020)(3) | Qualitative | Several countries (incl. Nigeria) | General | Maternal health Child health | Reduced availability of preventative services (e.g. immunisation, Sexual and Reproductive health (SRH), maternal and child health services), and services delivered by community health workers (CHW) were stopped due to movement restrictions. |
| Akseer N, Kandru G, Keats EC et al. (2020)(4) | Literature review | Low- and middle-income countries (LMICs) | Women and children | Nutrition | This review highlights key areas of concern for maternal and child nutrition in the context of the COVID-19 pandemic and provides a set of recommendations to address food insecurity, reduced income, restricted health services, and poor sanitation |
| Banke-Thomas A, Makwe CC, Balogun M et al. (2020)(5) | Cross-sectional | Nigeria | Women | Maternal health | Based on the experience of nine women with COVID-19, the cost doubled and tripled depending on the route of delivery and personal protective equipment (PPE) was the major justification for this. |
| Bizri AR, Khachfe HH, Fares MY et al. (2020)(6) | Cross-sectional | Lebanon | General | Child health | Routine immunisation was interrupted because of the social distancing rules and pre-planned second phase of the measles vaccination did not go ahead. |
| Coker M, Folayan MO, Michelow IC et al. (2020)(7) | Literature review | Sub-Saharan Africa | Children | Child health | Highlights the indirect impact of COVID-19 on major paediatric disease’s prevention and control efforts (e.g. malnutrition, malaria, tuberculosis, HIV). |
| Dinleyici EC, Borrow R, Safadi MAP et al. (2020)(8) | Literature review | Global (incl. LMICs) | Children | Child health | Disruptions of immunization strategies due to COVID-19 have been observed. The risk of missed vaccinations may pose a threat to concurrent or future outbreaks of vaccine preventable diseases; authors advocate for the prioritisation of immunization programs. |
| Enyama D, Chelo D, Noukeu Njinkui D et al. (2020)(9) | Cross-sectional | Cameroon | Children | Child health | Paediatricians report a drop in the use of health facilities (from 60.4% seeing more than 30 patients/week to 9.9%). The drop in attendance is explained not only because of the preventative measures imposed, but also from parent’s fear of visiting health facilities for the risk of infection. |
| Ijarotimi OA, Ubom AE, Olofinbiyi BA et al. (2020)(10) | Literature review | Nigeria | Women | Maternal health | Basic health care materials (e.g. PPE and COVID-testing) were lacking in the obstetrical services. |
| Lusambili AM, Martini M, Abdirahman F el a. (2020)(11) | Qualitative | Kenya | Women (refugees) | Maternal health | There was a decline in antenatal care (ANC) attendance and the level of home deliveries increased. Refugee women delayed seeking care due to fear of COVID-19 infection, increased financial burden and lack of inclusion in national health care policies. |
| Mahmassani D, Tamim H, Makki M, Hitti E. (2020)(12) | Cross-sectional | Lebanon | General | Child health | There was a significant drop in emergency department visits in the 3 months post COVID-19 diagnosis in the country when comparing with the prior 3 months, across all age groups, with paediatric patients (1-17 years) having the most significant decline. |
| Masresha BG, Luce Jr. R, Shibeshi ME. Et al. (2020)(13) | Cross-sectional | Africa (incl. Nigeria, Central African Republic (CAR), Chad, Democratic Republic of Congo (DRC), South Sudan, Burundi, Eritrea) | Children | Child health | Countries with lower vaccination coverage pre-COVID experience higher declines in the number of children vaccinated after the pandemic was declared. In countries with chronically low coverage (e.g. CAR, Chad, DRC) this large decline was not seen, authors hint that could be explained by the extent of the impact of COVID or the type of restrictions imposed. |
| Minckas N, Medvedev MM, Adejuyigbe EA et al. (2021)(14) | Modelling | LMICs | Newborns | Newborn health | A comparative risk analysis of the newborn deaths averted by sustained kangaroo mother care (KMC) practice in preterm newborns during the pandemic in LMICs. In their worst case scenario of 100% mother-infant SARS-CoV-2 transmission they estimated 1 950 newborn death from COVID-19 and 125 680 deaths averted with universal KMC. A 50% reduction in KMC coverage is estimated to result in 12 570 extra deaths and 25 140 with full disruption across 127 LMICs. |
| Moyo J, Madziyire G. (2020)(15) | Cross-sectional | Zimbabwe | Women | Maternal health | Use of telemedicine to manage gynaecological and obstetrical conditions during lockdown. It reports that was effective, could be used for triage, was accepted and that it managed to resolve the situation in 52.2% of the cases. |
| Ogundele IO, Alakaloko FM, Nwokoro CC, et al. (2020)(16) | Cross-sectional | Nigeria | Children | Child health | Overall cessation of elective surgeries (92% of centers) and continuation of the emergency ones, although noted a decline in the volume of these surgeries.  80% of centers did not offer telemedicine follow up. |
| Roberton T, Carter ED, Chou VB et al. (2020)(17) | Modelling | LMICs | Women and children | Child health  Maternal health | Authors estimated a substantial indirect mortality from the COVID-19 pandemic in LMICs, due to disruptions in essential maternal and child health interventions and access to food, leading to increased maternal and child deaths, due to increased prevalence of wasting and reduces coverage of certain life saving interventions. |
| Semaan A, Audet C, Huysmans E et al. (2020)(18) | Cross-sectional | Global (incl. LMICs) | Women and newborns | Maternal health  Newborn health | A global survey done for maternal and newborn health professionals (including LMICs) reports on the COVID-19 related measures taken within their health facilities; and list the several changes to service provision across the continuum of care. |
| Sherrard-Smith E, Hogan AB, Hamlet A et al. (2020)(19) | Modelling | Africa | General | Child health | If malaria control measures are severely disrupted, malaria deaths could more than double in 2020 when compared with 2019. In Nigeria alone, reducing case management for 6 months and delaying long lasting insecticidal net campaigns could result in 81,000 (44,000–119,000) additional deaths. |
| Zar HJ, Dawa J, Fischer GB et al. (2020)(20) | Literature review | LMICs | Children | Child health | Authors argue that the indirect effects of the pandemic on child health are significant in LMICs considering the increasing of poverty levels, suspension of school feeding programs, vaccination and reduced access to health care; combined with under-resourced health systems. |

Table 2. Characteristics and key findings of grey literature including supporting documents provided by key informants

| **Author and year** | **Type of publication** | **Setting** | **Target population** | **General topic** | **Summary** |
| --- | --- | --- | --- | --- | --- |
| Aborode et al. 2021 (21) | Perspective | LMICs, focus on Africa | Children | Nutrition | The COVID-19 pandemic has led to increases in food insecurities in low- and middle-income countries (LMICs), particularly sub-Saharan Africa and South Asia. Access to nutritious, affordable diets, improving maternal and early child nutrition programs (including the promotion and support of breastfeeding), reactivating early detection and treatment of wasting programs, maintaining school meal programs, and safeguarding nutritious diets for the most vulnerable households must be reinstituted to avert an excess of 40,000-2 million child deaths in LMICs. |
| Adamu et al. 2020 (22) | Perspective | Africa | Children | Child health | Childhood vaccinations in Africa are being disrupted by the COVID-19 pandemic. There is an increased risk of epidemics of vaccine-preventable diseases, which could increase child mortality. There is a need to identify and implement robust and context-specific measures to scale up immunisation programmes. The authors advocate for the use of systems thinking to enable this. |
| Adesunkanmi et al. 2020 (23) | Essay | Nigeria | Women | Maternal health | The cost of surgical and obstetric care was reviewed and compared pre- and during the COVID-19 pandemic. During the COVID-19 pandemic, the cost of surgical and obstetric care significantly increased as patients had to buy their own personal protective equipment (PPE) and pre-admission COVID-19 tests. |
| Ahmadi et al.  2020 (24) | Perspective | Afghanistan | Children | Child health | Prior to the pandemic, Afghanistan was struggling to eradicate polio due to the fragile health system, prolonged insecurity, misinformation and parental concerns. With COVID-19, polio has almost entirely been neglected. Polio cases are increasing in the country, particularly in polio-free provinces. After an initial lockdown, many businesses have been allowed to resume, but the mass polio vaccination campaign has not restarted. New cases of polio in children will surge if endemic regions remain unvaccinated or inaccessible. |
| Al Hosse and Edwards. 2020 (25) | News | Syria | Children | Nutrition | There is increasing acute and chronic childhood malnutrition in northwest Syria. One factor contributing is increasing poverty with rising food prices, an impact of COVID-19 and value of the Syrian pound. |
| Ameh et al. 2020 (26) | Pre-print (cross-sectional study) | Nigeria | Women and newborns | Maternal health  Newborn health | Providers (n=256) at reproductive, maternal, and newborn health facilities in Lagos, Nigeria, were surveyed to assess COVID19 preparedness and identify factors that significantly predict preparedness. 35.2% reported that reproductive, maternal and newborn health (RMNH) services were unavailable at some time during the pandemic. 84% were moderately or extremely concerned about the availability of PPE and related guidelines, and only 11.7% were extremely satisfied with their facility’s preparedness. |
| Busch-Hallen J et al.  2020 (27) | Correspondence | LMICs | Women and children | Maternal health  Child health  Nutrition | Breastfeeding is likely to decrease during COVID-19 due to limitations in the provision and use of health services and disruptions to the enabling environment. Depending on if the relative reduction in breastfeeding prevalence is hypothetically small (5%), moderate (10%), medium (25%), or severe (50%), this would result in 16 469 (small reduction), 32 139 (moderate reduction), 75 455 (medium reduction), and up to 138 398 (severe reduction) child deaths across 129 LMICs over a 1-year period, plus additional morbidity. |
| Cardwell and Ghazalian. 2020 (28) | Viewpoint | General | General | Nutrition | The COVID-19 pandemic is affecting the demand and the supply of international food assistance due to a global demand shock (increased need) plus supply shock (interruption to supply chains). The authors advocate for prioritised spending in aid-collection decisions by donor countries, exemption of food assistance from trade barriers (e.g. import tariffs) and flexibility by donor countries for implementing agencies by untying food assistance from domestic procurement and shipping restrictions. |
| CARE International. 2020 (29) | Report | General with focus on Cox’s Bazaar | Girls and adolescents | Maternal health | Antenatal care (ANC) fell by 65% use of services by adolescents between January and May 2020, which coincided with the March lockdown. Data also point to an increase in ANC attendance when lockdown ended. |
| CASS/UNICEF. 2020 (30) | Report | Democratic Republic of Congo (DRC) | Women and girls | Maternal health  Nutrition | Information gathered from different actors in DRC indicates high food prices, declining incomes, and the increase of the exchange rate limit women's ability to meet basic household needs, including food for children. There have been delays in usage of health services due to fear of COVID-19 or quarantine. ANC visits have been reduced, and there has been an increase in number of pregnancies and unsafe abortions. There has been an increase in family planning service use with women reporting a fear of becoming pregnant due to the cost of raising a child and increase in sexual activity among adolescents with school closures and financial difficulties leading to transactional sex. |
| Castro. 2020 (31) | Correspondence | Latin America and the Caribbean | Women and children | Maternal health  Child health | The COVID-19 pandemic in most Latin American and Caribbean countries has led to suspension or limitation of reproductive, maternal, newborn and child health (RMNCH) services. Cuba, Uruguay and Costa Rice have both the highest RMNCH index and dedicate the highest percentage of gross domestic product (GDP) to public spending on health, and have maintained access to all health services despite the pandemic. The authors argue this shows a need to strengthen RMNCH services rather than limiting them through increased financing, restoring and rebuilding essential health services and strengthening primary healthcare strategies. |
| Chatterjee A. 2020 (32) | Report | Middle East and Northern Africa (MENA) region (Incl. Iraq, Syria, Sudan, Yemen) | Children | Child health  Nutrition | Under-five child mortality in MENA may increase by approximately 40% due to healthcare disruptions secondary to COVID-19 including protracted reduction in the supply and demand of primary health care services, including case management of neonatal and childhood infections, child nutrition, essential care in the antenatal period and at birth and immunization. Prevention of these deaths requires resuming and maintaining essential health and nutrition services for children, pregnant women and mothers. Infection prevention and control (IPC) in health facilities needs to be strengthened. Community engagement is vital to build trust and promote appropriate health-seeking behaviours. |
| Chimhuya et al. 2021 (33) | Pre-print (interrupted time series analysis) | Zimbabwe | Newborns | Newborn health | This study examined trends in markers of neonatal care before and during the pandemic at two neonatal units in Zimbabwe and Malawi. The authors found that admissions did not change significantly after the first case of COVID-19 when considering this period as a whole, but there was a considerable decrease (~50%) in the number admissions in June to August 2020, coinciding with a nurses’ strike. |
| Dmello et al. 2020 (34) | Correspondence | LMICs | Women and children | Maternal health  Child health | Authors draw attention to the increased clinical complexity of maternity care in COVID, especially in countries with fragile health systems. Vertical responses to COVID might counteract years of advocacy and health system improvements for maternity care. |
| Dube et al. 2020 (35) | Field article | Cox's Bazaar - Bangladesh | Children | Nutrition | Adaptations were made to continue programmes such as the use of mid upper arm circumference (MUAC) only, mother-led MUAC screening, and change in the size of rations. |
| Edwards. 2020 (36) | Report | Global | Children | Child health  Nutrition | In a survey by Save the Children, 95% of households with children with a chronic health condition reported that the pandemic impacted their access to healthcare. 45% stated that their child was unable to access regular health and rehabilitation services. 96% of households with children with disabilities experienced reductions in access to healthcare. There has been an increase in food insecurity and acute and chronic malnutrition. |
| Elhadi et al. 2020 (37) | Brief communications | Libya | General | Maternal health | This small cross-sectional survey assesses the preparedness of obstetricians to manage the COVID‐19 pandemic. They identify a need to provide support for these healthcare workers. Obstetricians must be prepared with adequate training, sufficient PPE, and action plans. |
| Fore. 2020 (38) | Comment | LMICs | Children | Child health | This commentary from UNICEF proposes key areas of focus to overcome the negative impacts of the COVID19 pandemic on children's health: maintaining essential and life-saving maternal, newborn and child health (MNCH) services, prioritising funding and support for water, sanitation and hygiene (WASH), maintaining learning environments for children, engaging family members in child health and improving sexual and gender based violence (SGBV) services are women and children are more vulnerable to SGBV in the pandemic. |
| Fore et al. 2020 (39) | Comment | LMICs | Children | Nutrition | This comment presents the adverse impacts of COVID-19 on malnutrition in children, calling for immediate actions including ensuring access to food, improving maternal and child nutrition, and detecting child wasting. |
| Francis and Pegg. 2020 (40) | Editorial | Niger Delta, Nigeria | Children | Nutrition | A weekly nutritional program in the Niger Delta region of Nigeria provides an example of how to prevent further malnutrition in rural children by adapting the program to social distancing measures of the COVID-19 era. |
| Global Financing Facility. 2020 (41–47) | Country briefs | Central African Republic (CAR), DRC, Niger, Nigeria, Somalia, South Sudan, Yemen | Women and children | Maternal health  Child health | Factors affecting utilisation of essential services during COVID-19 include demand-side factors (restrictions of movement, loss of income, concern of contracting COVID-19), and supply side factors (supply chain disruptions, staff losses, COVID-19 cases taking up health service capacity). Estimated impacts are children unable to access antibiotics for pneumonia, children not receiving diphtheria-tetanus-pertussis (DTP) vaccination, reductions in numbers of women with access to family planning services and resulting rises in maternal and child mortality. |
| Govender et al. 2020 (48) | Perspective | Eastern and southern Africa | Children and youth (<25 years old) | Child health | The COVID-19 pandemic and mitigation measures to stop the spread of the virus have created specific challenges for children and young people (CYP) living in Eastern and Southern Africa. Continued access to healthcare, education, financial opportunities, and food must prioritize at-risk CYP to prevent further marginalization. |
| Graham et al. 2020 (49) | Commentary | LMICs | Women and newborns | Maternal health  Newborn health | Both funding to maintain essential services and urgent action plans for COVID-19 are needed to mitigate the adverse impact of COVID-19 on maternal and newborn health outcomes in LMICs. |
| Headey et al. 2020 (50) | Comment | LMICs | Children | Child health | The authors argue that without adequate action, the profound impact of the COVID-19 pandemic on early life nutrition could have intergenerational consequences for child growth and development and lifelong impacts on education, chronic disease risks, and overall human capital formation. |
| Hobday et al. 2021 (51) | Comment | Humanitarian settings | Women | Maternal health | This article writes in favour of distributing misoprostol, a medication that helps prevent post-partum haemorrhage, widely to women who cannot access healthcare facilities during the COVID-19 pandemic. The authors cite previous distributions during the Ebola outbreak and the Nepalese earthquake as proof that mass distribution can be done even amid healthcare disruptions. |
| Hrynick et al. 2020 (52) | Review | LMICs | General | Maternal health  Child health | Reduced care coverage is likely to lead to an additional 57,000 maternal deaths. Neonatal deaths are also expected to increase. Reductions in contraceptive use may lead to unplanned pregnancies. There may be an additional 1.2 million child deaths due to malnutrition and vaccine-preventable diseases. |
| ICRC. 2020 (53) | News | Nigeria | Children | Nutrition | Reports a surge in malnutrition cases in one hospital. |
| ICRC. 2020 (54) | News | Somalia | Women and children | Child health  Maternal health | Visits to Somali Red Crescent health clinics by children under five and pregnant women reduced by more than 50 per cent from approximately 181,000 medical consultations in the first seven months of 2019 to nearly 83,000 in the same period of 2020. There has been reduced vaccination coverage of children, rises in cases of acute watery diarrhoea and measles. |
| Immap/DFS USAID. 2020 (55) | Report | Syria | General | Nutrition | The prevalence of chronic malnutrition for children 6-59 months increased from 19 to 34% between May 2019 and October 2020. The number of children with severe acute malnutrition (SAM) in need to hospitalisation increased by over 70% since March 2020 when compared with the same period in 2019. Between 20 and 37% of mothers were reportedly malnourished in December 2020 (proxy prevalence across the region is 11%). |
| International Development Committee. 2020 (56) | Report | Humanitarian crises in developing countries | General | Nutrition  Child health | The International Development Committee conducted an enquiry where they found widespread disruptions to routine vaccination programmes with 70% of countries experiencing stalling of routine immunisation programmes, rising unemployment leading to fears of accessing food and lockdowns resulting in increased gender-based violence. |
| Inter-Sector Coordination Group Gender Hub. 2020 (57) | Report | Cox's Bazaar - Bangladesh | General | Nutrition  Maternal health | Rohingya refugees and host communities reported difficulty in accessing services including maternal health services and support for gender based violence (GBV). Reductions in ability to buy food due to income disruptions, and difficulty accessing food assistance was reported. Increases in GBV has been reported, with a gendered power dynamics putting women at risk. |
| IRC. 2020 (58) | Report | South Sudan | Children | Nutrition | This report provides recommendations on how to simplify treatment of acute malnutrition, including scaling up community health workers (CHW), promote outpatient care to reduce overcrowding and COVID-19 spread, reduce to two body measurements (checking oedema and assessing MUAC) and promote caregiver MUAC measurement, and give all children diagnosed with acute malnutrition the same therapeutic food. |
| Janvrin and McKay. 2020 (59) | Report | Humanitarian contexts | Women and newborns | Maternal health  Newborn health | Maternal, Newborn, and Reproductive health (MNRH) priorities were not included in Country Preparedness Plans during the initial COVID-19 outbreak in January 2020. A consultation in October 2020 identified challenges including decreased demand and supply of quality MNRH services, interrupted data collection systems and diversion of finances towards COVID-19. Adaptations included social distancing measures in clinics, online data collection and online trainings. Recommendations include strengthening health workforce and delivery, community level MNRH services and guidelines/tools, and improving coordination. |
| Kabir et al. 2020 (60) | Letter to editor | Low- income countries (LIC) - humanitarian programmes | Children | Nutrition | This letter argues that COVID-19 adversely affects the economies worldwide with particular impact on child nutrition, especially those in poverty. The authors highlight concern of the impact on global economies leading to decreased funding for humanitarian programs in nutrition. |
| Khorsandi. 2020 (61) | News | Yemen | Children | Nutrition | This article highlights the risk of worsening famine in Yemen. Due to the lack of funds, the organisation had to cut rations affecting 9 million people. |
| Laouan. 2020 (62) | Report | West Africa (Niger, Nigeria, Mali, Cameroon) | Girls | Maternal health | In a survey, women reported that the health centres were functional but limited however officially offered prenatal consultations. There was decreased visiting of health centres due to fear of contracting COVID-19, and the increased waiting time due to social distancing measures. There were some reports of women not being allowed to attend health centres by their husband due to rumours of health professionals spreading COVID-19. |
| Martinez et al. 2020  (63) | Report | Afghanistan | Children | Child health | House to house polio outbreak response campaigns were aborted for 5 months due to COVID-19, resulting in increased numbers of children susceptible especially in insurgency-held areas. An increase in polio cases was seen during 2020. |
| McClure et al. 2020 (64) | Correspondence | LMICs | Women and newborns | Maternal health  Newborn health | Responds to an analysis by Robertson et al estimating the indirect effects of COVID-19 on maternal and child mortality in LMICs with use of the Lives Saved Tool (LiST). Highlights the likely under-estimation of deaths as stillbirths are omitted in Roberston et al.’s analysis. |
| McKernan. 2020 (65) | News | Yemen | General | Nutrition | Aid cuts and rising food prices are putting Yemen at risk of famine. Assessing the impact of COVID is difficult due to lack of testing facilities and routine data. |
| Mellis. 2020 (66) | Commentary | Africa | Children | Child health | According to the epidemic models created by the authors, the benefit of childhood immunizations outweighs the COVID-19 risk. Vaccination should be continued with the appropriate use of PPE, good hygiene, and physical distancing measures. |
| Menendez et al. 2020 (67) | Comment | LMICs | Women and children | Maternal health  Child health | This commentary highlights limitations of the Robertson et al. modelling study on indirect effects of the COVID-19 pandemic on maternal and child health, while highlighting an additional example of disruptions in antenatal care in some African countries. |
| MSF. 2020 (68) | Report | Afghanistan, Syria, Yemen, Somalia, Niger, Mali | General | Maternal health  Nutrition | In Yemen, fear of contracting COVID-19 in a health facility or of being stigmatised in case of a positive test result deterred people from seeking medical care. In Yemen, Niger and Mali, increases in admissions for severe malnourishment in child feeding centres were seen from late August 2020. In Afghanistan, maternity admissions decreased by around 50%. There were maternity staff shortages because of quarantine. Furthermore, a reduction in contraceptive consultations could be offered possibly leading to an increase in unwanted pregnancies. |
| Murewanhema et al.  2020 (69) | Perspective | Zimbabwe | General | Maternal health  Child health | This publication reports on disruptions to essential maternal and child health (MCH) and sexual reproductive health (SRH). ANC was neglected, leading to women with obstetric complications presenting late or in labour. There have been reported shortages in contraceptives at clinics. Many vaccination clinics were stopped in the beginning of the lockdown, meaning that many children likely missed doses. |
| Mustafa and Green. 2020 (70) | Editorial | Africa | Children | Child health | The authors discuss how many common infectious diseases in Africa present with similar symptoms to COVID-19 (malaria, viral respiratory tract infections, pneumonia, etc.) and at present comprise more disease burden than COVID-19. There is a risk of reduced quality and provision of paediatric care if hospitals become overwhelmed with COVID-19 patients. |
| Nelson. 2020 (71) | Newsdesk | LMIC | General with focus on children | Child health | Catch-up immunization campaigns will be necessary to account for disruptions in vaccine delivery across LMICs due to the COVID-19 pandemic. |
| Nghochuzie et al.  2020 (72) | Perspective | Africa | General with focus on children | Child health | Although SARS-CoV-2 is less pathogenic in young children, this population has the highest burden of malaria. The authors argue that neglecting efforts to control malaria in favour of COVID-19 could prove catastrophic for global health, particularly in Africa. |
| Nwafor et al. 2020 (73) | Pre-print (cross-sectional study) | Nigeria | Women | Maternal health | The authors conducted a questionnaire-based cross-sectional study among 456 pregnant women attending prenatal care in Nigeria during COVID-19- related lockdown. The authors found that depression (45.2%), anxiety (37.5%), and stress (56.8%) symptoms were relatively common among pregnant women. |
| OCHA. 2020 (74) | Report | Syria | General | Nutrition | Increasing malnutrition is seen across northwest Syria. There was a 55% increase in cases of SAM and an increase of 5% in stunting of children aged 6-59 months in February 2021 compared to January 2020. Women and girls, including pregnant and lactating women, are disproportionately affected by malnutrition. Several mothers were refusing to perform caregiver MUAC screening on their children due to the spread of COVID-19. Funding constraints were faced by several Nutrition Cluster partner, limiting ability to scale-up responses and get to populations in inaccessible locations. |
| Ogundele et al. 2020 (75) | Commentary | Nigeria | Children | Child health | This commentary highlights the importance of maintaining essential services such as childhood vaccination in times of emergency. The COVID-19 outbreak has posed a new hindrance to vaccination activities in Nigeria and across Sub-Saharan Africa with associated threat to surveillance of vaccine-preventable diseases. The authors advocate for high levels of vaccination coverage to be a priority for all health systems during COVID-19. |
| Ogunkola et al. 2021 (76) | Letter to editor | Sub-Saharan Africa | Women | Maternal health | This letter to the editor warns of increasing maternal morbidity and mortality in Sub-Saharan Africa during the COVID-19 pandemic due to shifts in funding, closures of sexual and reproductive health clinics, and disruptions of an already fragile health system infrastructure. |
| Penn-Kekana. 2020 (77) | Blog | Several countries (incl. Bangladesh and Nigeria) | Women | Maternal health  Newborn health | This blog draws on experiences shared by MNCH practitioners from different countries, including Bangladesh and Nigeria.. ANC attendance dropped in all settings, with women arriving at facilities later with complications (e.g. eclampsia). Pregnant women and providers faced delays in getting to facilities due to COVID-19- related lockdowns, curfews, public transport shut-downs, and ambulances being used for COVID-19 patients. Respectful maternal and newborn care may be compromised, such as by not allowing birth partners to attend. Adaptations included social distancing measures for ANC clinics. |
| Physicians Across Continents. 2020 (78) | PowerPoint presentation | Syria | Women | Nutrition | In Northwest Syria, a survey from May 2020 reports that 34.2% of women were providing exclusive breastfeeding and 50.3% were continuing breastfeeding. |
| Rahimov et al. 2020(79) | Field article | Cox's Bazaar - Bangladesh | General | Nutrition | In Cox’s Bazar, adaptations were made to continue programmes through integration of malnutrition screening in the vitamin A supplementation campaigns. |
| Ramoni. 2020 (80) | News | Nigeria | Women | Maternal health | Women experienced a reduction in ANC appointments and in care given when attending ANC. Social distancing measures and increased IPC measures were put in place. Some hospitals used telemedicine for ANC. |
| Reuters. 2020 (81) | News | Yemen | Women and children | Nutrition | COVID-19 was a driver for worsening malnutrition in Yemen. In 2020, cases of acute malnutrition in children under five increased about 10% to more than 500,000. Cases of children with severe acute malnutrition rose by 15.5% to 98,000. At least 250,000 pregnant or breastfeeding women need malnutrition treatment. Several nutrition services in Yemen are closing due to funding shortages. |
| Richards et al. 2020 (82) | Report | Chad, Nigeria | Children | Nutrition | This report highlights the level of food insecurity seen during COVID-19 in households in several countries and the impact on children. In Chad and Nigeria 77 and 78% respectively of surveyed households experienced food insecurity during the pandemic. |
| Riley et al. 2020 (83) | Comment | LMICs | Women and newborns | Maternal health  Newborn health | The authors present two scenarios of how the COVID-19 pandemic could disrupt sexual and reproductive health service provision in LMICs, and illustrate the impact of these changes on the number of unintended pregnancies, unsafe abortions, and maternal and newborn deaths. |
| Roberts. 2020 (84) | Comment | DRC | General | Child health | Scientific advisory group for Emergencies (SAGE) recommended that countries temporarily suspend all preventive mass-vaccination campaigns, including for measles, in March 2020. This translates as 78 million children missing their vaccination. DRC, where there was a large measles outbreak at the time of writing, continued its’ outbreak response however the virus is expected to rebound given global reductions in coverage. |
| Roberts. 2020 (85) | Comment | LMICs | Children | Child health | Due to social distancing orders, the suspension of mass vaccination campaigns for highly contagious diseases, including measles and polio, place children, especially those who are malnourished, at risk. |
| Save the Children. 2020 (86) | Press release | Humanitarian crises | Children | Nutrition | An estimated total of 60,237,000 children will need humanitarian assistance in Yemen, Ethiopia, DRC, Afghanistan, Sudan, Syria, Pakistan, and Nigeria. Of the roughly 117.7 million children who need support in 2021, 60 million live in just eight countries, with Yemen, Ethiopia, and the DRC accounting for more than 10 million children each. |
| Save the Children. 2020 (87) | Report | Somalia | Women and children | Nutrition  Child health | A survey by Save the Children highlighted that COVID-19 had reduced health seeking and access for women and children with barriers including lack of medicine at facilities, movement restrictions and lack of knowledge of where to find services. Food insecurity has increased with 75% of respondents reporting reducing quantity and quality of food consumption. |
| Save the Children. 2020 (88) | News | Yemen | Children | Nutrition  Child health | There has been a 74% reduction in services for malnutrition in Yemen. There has also been a 48% reduction in infant vaccinations. Difficulties have been finding funding, with only 18% of the required assistance available. Staff and supplies have been diverted to COVID-19 and a cholera outbreak. |
| Stein et al. 2020 (89) | News | LMICs | Women and newborns | Maternal health  Newborn health | Access to quality maternal health care is likely to be reduced, and disrupted maternal health care may lead to significant increases in maternal and newborn deaths and in stillbirths. The authors call for countries to take actions to maintain continuity of maternal and newborn health (MNH) services including using COVID-19 response funds for maintaining MNH services. |
| The Global Fund. 2020 (90) | Report | Sub-Saharan Africa | Children | Child health  Maternal health | Children may not be able to access timely treatment for malaria due to health services overwhelmed with COVID-19, or facing healthcare worker shortages. Healthcare workers may be unable or reluctant to test children with fevers due to risk of COVID-19 exposure, difficulty travelling, or sickness. Lockdown and travel restrictions are barriers to pregnant women with HIV accessing health services. |
| UN Country Office Syria. 2020 (91) | PowerPoint presentation | Syria | Women and children | Maternal health  Newborn health  Child health | Reproductive health services were disrupted, and prenatal care visits were limited which increased the health risks for pregnant women and newborns. Vaccination programmes decreased by around 40%. |
| UNFPA. 2021 (92) | Report | Syria | Women and girls | Maternal health | A report from Northwest Syria refers that where certain solutions were in place, the number of consultations increased. Telemedicine was provided. There was an overall decline in women visiting health facilities, with a decrease happening at the beginning of the pandemic. A survey and separate assessment found that 93% and 95% of women respectively delivered in a health facility in Idlib between April and June 2020. In northwest Syria, access difficulties were related to the visitor restrictions inside the hospitals whereby it was more difficult for women to go alone. |
| UNICEF. 2020(93) | Report | Cox’s Bazaar, Bangladesh | Children | Child health  Nutrition | As of November 2020, catch up vaccinations have been done with 89% of planned sessions held in the camps. Until October 20, there was an upward trend in admission of children under-5 with SAM. |
| UNICEF. 2020 (94) | PowerPoint presentation | Somalia | Women and children | Maternal health  Child health | In Somalia, attendance of first ANC visit decreased 5% between March and May 2020 but increased 2% when compared with 2019. There was no observed temporary decrease regarding attendance of the fourth ANC visit until September 2020. There was no decrease in facility-based deliveries due to COVID-19. There was a 4-5% decrease of EPI services between March and May 2020 and this has recovered from June 2020 onwards. |
| UNICEF. 2020 (95) | News | Yemen | Children | Nutrition | In parts of Yemen, 20% of children under five are estimated to be acutely malnourished and in urgent need of treatment. Factors worsening the situation include the impact of COVID-19 and funding shortfalls. |
| UNICEF. 2020 (96) | PowerPoint presentation | Zimbabwe | General | Nutrition | Situation analysis of continuity of critical health and nutrition services until end of November 2020. |
| Walker and Chandir.  2021 (97) | Correspondence | Africa | Children | Child health | The authors respond to a study by Abbas et al. (Oct 2020), which had modelled a 6-month COVID-19 risk period for disruptions in routine health services. They highlight that immunization programs have been severely disrupted, and better estimates of the numbers of missed doses of vaccines in children are needed to plan and implement catch-up programs. |
| Wangamati and Sundby. 2020 (98) | Commentary | Kenya | Women | Maternal health | Relocation of services to COVID-19 slowed the service delivery of maternal health. There was a decrease in attendance of pregnant women at facilities as they did not know where to go. There was a lack of PPE for the existing maternal health services. |
| West. 2020 (99) | Report | Humanitarian settings | General | Child health  Nutrition | Mass vaccination campaigns were advised to be stopped or delayed in 54 countries, meaning many children have missed doses. Supply chain and human resource constraints from COVID-19 have made epidemic response to measles challenging in DRC, Chad and CAR. COVID-19 has increased food insecurity, with increased inpatient therapeutic feeding centre admissions in Yemen, Niger, and Mali. COVID-19 has increased borrowing for domestic budgets worldwide, and it is expected that donor countries will have less to spend on international aid. |
| World Health Organization. 2020 (100) | Report | Sub-Saharan Africa | General | Child health | This report models scenarios of potential disruption of malaria control measures due to COVID-19 in malaria-endemic countries. In the most severe case (no insecticide treated net (ITN) campaigns, both continuous ITN distributions and access to effective antimalarial treatment reduced by 75%), malaria deaths may increase by 20-200% of baseline. The majority of malaria deaths occur in children under five. |
| World Vision. 2020 (101) | Report | Venezuela | Children | Nutrition | 35% of respondents reported food shortages due to the COVID-19 pandemic. The report calls on governments to safely adapt critical food-assistance and nutrition programmes, strengthen health systems and ensure continuity of essential nutrition services. Other actors including donors should scale up response efforts to tackle malnutrition. |
| The Lancet Global Health. 2020 (102) | Editorial | Sub-Saharan Africa | General | Nutrition | COVID-19 has worsened food insecurity and malnutrition due to economic instability and supply chain disruptions. There is a potential for the COVID-19 pandemic to be compounded by a pandemic of undernutrition, with women and children worst affected. Preparing for this requires increased donor funding to the UN COVID-19 Global Humanitarian Response Fund, as well as balancing COVID-19 safety measures (such as preventing transport and trade) with risks to food supply chains. |

1. Abbas K, Procter SR, van Zandvoort K, Clark A, Funk S, Mengistu T, et al. Routine childhood immunisation during the COVID-19 pandemic in Africa: a benefit–risk analysis of health benefits versus excess risk of SARS-CoV-2 infection. Lancet Glob Heal. 2020;8(10):e1264–72.

2. Abdulah DM, Abdulla BMO, Liamputtong P. Psychological response of children to home confinement during COVID-19: A qualitative arts-based research. Int J Soc Psychiatry. 2020/11/14. 2020;20764020972439.

3. Ahmed SAKS, Ajisola M, Azeem K, Bakibinga P, Chen Y-F, Choudhury NN, et al. Impact of the societal response to COVID-19 on access to healthcare for non-COVID-19 health issues in slum communities of Bangladesh, Kenya, Nigeria and Pakistan: results of pre-COVID and COVID-19 lockdown stakeholder engagements. BMJ Glob Heal. 2020;5(8).

4. Akseer N, Kandru G, Keats EC, Bhutta ZA. COVID-19 pandemic and mitigation strategies: implications for maternal and child health and nutrition. Am J Clin Nutr. 2020/06/20. 2020;112(2):251–6.

5. Banke-Thomas A, Makwe CC, Balogun M, Afolabi BB, Alex-Nwangwu TA, Ameh CA. Utilization cost of maternity services for childbirth among pregnant women with coronavirus disease 2019 in Nigeria’s epicenter. Int J Gynaecol Obs. 2020/10/25. 2020;

6. Bizri AR, Khachfe HH, Fares MY, Musharrafieh U. COVID-19 Pandemic: An Insult Over Injury for Lebanon. J Community Health. 2020;

7. Coker M, Folayan MO, Michelow IC, Oladokun RE, Torbunde N, Sam-Agudu NA. Things must not fall apart: the ripple effects of the COVID-19 pandemic on children in sub-Saharan Africa. Pediatr Res. 2020 Sep;1–9.

8. Dinleyici EC, Borrow R, Safadi MAP, van Damme P, Munoz FM. Vaccines and routine immunization strategies during the COVID-19 pandemic. Hum Vaccin Immunother. 2020/08/28. 2020;1–8.

9. Enyama D, Chelo D, Noukeu Njinkui D, Mayouego Kouam J, Fokam Djike Puepi Y, Mekone Nkwele I, et al. Impact of the COVID-19 pandemic on pediatricians’ clinical activity in Cameroon. Arch Pediatr. 2020;

10. Ijarotimi OA, Ubom AE, Olofinbiyi BA, Kuye-Kuku T, Orji EO, Ikimalo JI. COVID-19 and obstetric practice: A critical review of the Nigerian situation. Vol. 151, International Journal of Gynecology and Obstetrics. John Wiley and Sons Ltd.; 2020. p. 17–22.

11. Lusambili AM, Martini M, Abdirahman F, Asante A, Ochieng S, Guni JN, et al. “We have a lot of home deliveries” A qualitative study on the impact of COVID-19 on access to and utilization of reproductive, maternal, newborn and child health care among refugee women in urban Eastleigh, Kenya. J Migr Heal. 2020;1–2.

12. Mahmassani D, Tamim H, Makki M, Hitti E. The impact of COVID-19 lockdown measures on ED visits in Lebanon. Am J Emerg Med. 2020/12/16. 2020;

13. Masresha BG, Luce Jr. R, Shibeshi ME, Ntsama B, N’Diaye A, Chakauya J, et al. The performance of routine immunization in selected African countries during the first six months of the COVID-19 pandemic. Pan Afr Med J. 2020;37(Suppl 1):12.

14. Minckas N, Medvedev MM, Adejuyigbe EA, Brotherton H, Chellani H, Estifanos AS, et al. Preterm care during the COVID-19 pandemic: A comparative risk analysis of neonatal deaths averted by kangaroo mother care versus mortality due to SARS-CoV-2 infection. EClinicalMedicine. 2021;000:100733.

15. Moyo J, Madziyire G. Use of telemedicine in obstetrics and gynaecology in zimbabwe during a lockdown period. Pan Afr Med J. 2020;35(Supplement 2):1–4.

16. Ogundele IO, Alakaloko FM, Nwokoro CC, Ameh EA. Early impact of COVID-19 pandemic on paediatric surgical practice in Nigeria: A national survey of paediatric surgeons. BMJ Paediatr Open. 2020 Sep;4(1):732.

17. Roberton T, Carter ED, Chou VB, Stegmuller AR, Jackson BD, Tam Y, et al. Early estimates of the indirect effects of the COVID-19 pandemic on maternal and child mortality in low-income and middle-income countries: a modelling study. Lancet Glob Heal. 2020;8(7):e901–8.

18. Semaan A, Audet C, Huysmans E, Afolabi B, Assarag B, Banke-Thomas A, et al. Voices from the frontline: findings from a thematic analysis of a rapid online global survey of maternal and newborn health professionals facing the COVID-19 pandemic. BMJ Glob Heal. 2020/06/27. 2020;5(6).

19. Sherrard-Smith E, Hogan AB, Hamlet A, Watson OJ, Whittaker C, Winskill P, et al. The potential public health consequences of COVID-19 on malaria in Africa. Nat Med. 2020/08/10. 2020;26(9):1411–6.

20. Zar HJ, Dawa J, Fischer GB, Castro-Rodriguez JA. Challenges of COVID-19 in children in low- and middle-income countries. Paediatr Respir Rev. 2020/07/14. 2020;35:70–4.

21. Aborode AT, Ogunsola SO, Adeyemo AO. A crisis within a crisis: Covid-19 and hunger in African children. American Journal of Tropical Medicine and Hygiene. 2021 Jan;30–1.

22. Adamu AA, Jalo RI, Habonimana D, Wiysonge CS. COVID-19 and routine childhood immunization in Africa: Leveraging systems thinking and implementation science to improve immunization system performance. Vol. 98, International Journal of Infectious Diseases. Elsevier B.V.; 2020. p. 161–5.

23. Adesunkanmi AO, Ubom AE, Olasehinde O, Fasubaa OB, Ijarotimi OA, Adesunkanmi ARK, et al. Impact of COVID-19 on the cost of surgical and obstetric care: experience from a Nigerian teaching hospital and a review of the Nigerian situation. Pan Afr Med J. 2020;37(Suppl 1):15.

24. Ahmadi A, Essar MY, Lin X, Adebisi YA, Lucero-Prisno DE. Polio in Afghanistan: The current situation amid COVID-19. Am J Trop Med Hyg. 2020;103(4):1367–9.

25. Al Hosse M, Edwards M. Inside the childhood hunger ‘emergency’ in Syria’a Idlib. The New Humanitarian. 2020 Oct;

26. Ameh CA, Banke-Thomas AA, Balogun M, Makwe CC, Afolabi B. Reproductive Maternal and Newborn Health providers assessment of facility preparedness and its Determinants during the COVID-19 pandemic in Lagos, Nigeria. 2020.

27. Busch-Hallen J, Walters D, Rowe S, Chowdhury A, Arabi M. Impact of COVID-19 on maternal and child health. Vol. 8, The Lancet Global Health. Elsevier Ltd; 2020. p. e1257.

28. Cardwell R, Ghazalian PL. COVID-19 and international food assistance: policy proposals to keep food flowing. Spec Sect Pandemics Sustain. 2020;135.

29. CARE. Girl-Driven Change. Meeting the needs of Adolescent Girls During COVID-19 and Beyond. 2020.

30. Social Sciences Analytics Cell (CASS). The impacts of the COVID-19 outbreak response on women and girls in the Democratic Republic of the Congo. 2020.

31. Castro A. Maternal and child mortality worsens in Latin America and the Caribbean. Lancet. 2020;396:e85.

32. Chatterjee A. The Potential Impact of Health Care Disruption on Child Mortality in the Middle East and North Africa due to COVID-19. 2020 Jun.

33. Chimhuya S, Neal SR, Chimhini G, Gannon H, Cortina-Borja M, Crehan C, et al. Indirect impacts of the COVID-19 pandemic at two tertiary neonatal units in Zimbabwe and Malawi: an interrupted time series analysis. medRxiv. Cold Spring Harbor Laboratory Press; 2021 Jan.

34. Dmello BS, Housseine N. Impact of COVID-19 on maternal and child health. 2020;

35. Dube BT, Chelang M, Mustaphi P, Harlass S, Bourdaire J, Singh K, et al. Adaptations to CMAM programming in Cox’a Bazar in the context of the COVID-19 pandemic. Field Exchange - Emergency Nutrition Network ENN. 2020;(63):57–60.

36. Edwards J. Protect a Generation. The impact of COVID-19 on children’s lives. 2020.

37. Elhadi M, Msherghi A, Elgzairi M, Alsuyihili A, Elkhafeefi F, Bouhuwaish A, et al. Assessment of the preparedness of obstetrics and gynecology healthcare systems during the COVID-19 pandemic in Libya. Int J Gynaecol Obstet. 2020;150:406–24.

38. Fore HH. A wake-up call: COVID-19 and its impact on children’s health and wellbeing. Vol. 8, The Lancet Global Health. Elsevier Ltd; 2020. p. e861–2.

39. Fore HH, Dongyu Q, Beasley DM, Ghebreyesus TA. Child malnutrition and COVID-19: the time to act is now. Vol. 396, The Lancet. Lancet Publishing Group; 2020. p. 517–8.

40. Francis NN, Pegg S. Socially distanced school-based nutrition program under COVID 19 in the rural Niger Delta. Extr Ind Soc. 2020/04/24. 2020;7(2):576–9.

41. Global Financing Facility. Preserver les Services de Sante Essentiels Pendant la Pandemie de COVID-19 - Republique Centrafricane. 2020 May.

42. Global Financing facility. Preserver les Services de Sante Essentiels Pendant la Pandemie de COVID-19 - Republique Democratique du Congo. 2020 May.

43. Global Financing Facility. Preserver les Services de Sante Essentiels Pendant la Pandemie de COVID-19 - Niger. 2020 May.

44. Global Financing Facility. Preserve Essential Health Services During the COVID-19 Pandemic - Nigeria. 2020 May.

45. Global Financing Facility. Preserve Essential Health Services During the COVID-19 Pandemic - Somalia. 2020 May.

46. Global Financing Facility. Preserve essential Health Services During the COVID-19 Pandemic - South Sudan. 2020 May.

47. Global Financing Facility. Preserve Essential Health Services during the COVID-19 Pandemic - Yemen. 2020 May.

48. Govender K, Cowden RG, Nyamaruze P, Armstrong RM, Hatane L. Beyond the Disease: Contextualized Implications of the COVID-19 Pandemic for Children and Young People Living in Eastern and Southern Africa. Front Public Heal. 2020 Oct;8:504.

49. Graham WJ, Afolabi B, Benova L, Campbell OMR, Filippi V, Nakimuli A, et al. Protecting hard-won gains for mothers and newborns in low-income and middle-income countries in the face of COVID-19: Call for a service safety net. BMJ Global Health. 2020 Jun;5(6):2754.

50. Headey D, Heidkamp R, Osendarp S, Ruel M, Scott N, Black R, et al. Impacts of COVID-19 on childhood malnutrition and nutrition-related mortality. Vol. 396, The Lancet. Lancet Publishing Group; 2020. p. 519–21.

51. Hobday K, Prata N, Hulme J, Homer CS. Preventing post-partum haemorrhage at home during COVID-19: what are we waiting for? Lancet Glob Heal. 2021 Jan;9(3):e245–6.

52. Hrynick T, Ripoll S, Carter S. Review: Broader Health Impacts of Vertical Responses to COVID-19 in Lon- and Middle- Income Countries (LMICs). Brighton; 2020.

53. International Committee of the Red Cross. Nigeria: Surge in Malnutrition Cases Raises Fear of Looming Disaster. International Committee of the Red Cross. 2020 Dec;

54. ICRC. Somalia: Decline in primary health care visits and childhood vaccinations during COVID-19 . 2020 Aug;

55. iMMAP/DFS COVID-19. Situational Analysis. Update#4 - Syria. 2020.

56. International Development Committee. Covid-19 in developing countries: secondary impacts. 2020.

57. Inter-Sector Coordination Group (ISCG) Gender Hub. In the shadows of the pandemic gendered impact of covid19 on rohingya and host communities. 2020.

58. IRC. Continuing care during COVID-19: Adopting Life-Saving Approaches to Treat Acute Malnutrition. 2020.

59. Janvrin A, McKay G. READY: Global readiness for major disease outbreak response. Maternal, Newborn, and reproductive health and COVID-19: Adaptations, successes, challenges, and next steps. An expert consultation. 2020.

60. Kabir M, Saqib MAN, Zaid M, Ahmed H, Afzal MS. COVID-19, economic impact and child mortality: A global concern. Clin Nutr. 2020/06/06. 2020;39(7):2322–3.

61. Khorsandi P. World Food Programme warns of worsening famine in Yemen . World Food Program. 2021 Jan;

62. Laouan FZ. Rapid Gender Analysis COVID-19. West Africa-April 2020. 2020.

63. Martinez M, Akbar IE, Wadood MZ, Shukla H, Jorba J, Ehrhardt D. Progress Toward Poliomyelitis Eradication - Afghanistan, January 2019-July 2020. MMWR Morb Mortal Wkly Rep. 2020;69(40):1464–8.

64. McClure EM, Kinney M V., Leisher SH, Nam SL, Quigley P, Storey C, et al. Impact of COVID-19 on maternal and child health. Vol. 8, The Lancet Global Health. Elsevier Ltd; 2020. p. e1258.

65. McKernan B. Yemen: in a country stalked by disease, Covid barely registers. The Guardian. 2020 Nov;

66. Mellis C. Sustaining routine childhood immunisations during COVID-19 in Africa. J Paediatr Child Health. 2020 Oct;jpc.15228.

67. Menendez C, Gonzalez R, Donnay F, Leke RGF. Avoiding indirect effects of COVID-19 on maternal and child health. Vol. 8, The Lancet Global Health. Elsevier Ltd; 2020. p. e863–4.

68. MSF. Responding to COVID-19. Global Accountability Report 2. June to August 2020. 2020.

69. Murewanhema G, Makurumidze R. Essential health services delivery in Zimbabwe during the COVID-19 pandemic: perspectives and recommendations. Pan Afr Med J. 2020;35(Suppl 2):143.

70. Mustafa F, Green RJ. The implications of COVID-19 for the children of Africa. South African Med J. 2020 Jun;110(6).

71. Nelson R. COVID-19 disrupts vaccine delivery. Lancet Infect Dis. 2020 May;20(5):546.

72. Nghochuzie NN, Olwal CO, Udoakang AJ, Amenga-Etego LN-K, Amambua-Ngwa A. Pausing the Fight Against Malaria to Combat the COVID-19 Pandemic in Africa: Is the Future of Malaria Bleak? Front Microbiol. 2020 Jun;11:1476.

73. Nwafor JI, Okedo-Alex IN, Ikeotuonye AC. Prevalence and predictors of depression, anxiety and stress symptoms among pregnant women during COVID-19-related lockdown in Abakaliki, Nigeria. 2020.

74. OCHA. Recent Developments in Northwest Syria. Situation Report No. 21. 2020 Oct.

75. Ogundele OA, Omotoso AA, Fagbemi AT. COVID-19 outbreak: a potential threat to routine vaccination programme activities in Nigeria. Hum Vaccines Immunother. 2020;

76. Ogunkola IO, Adebisi YA, Imo UF, Odey GO, Esu E, Lucero-Prisno DE. Impact of COVID-19 pandemic on antenatal healthcare services in Sub-Saharan Africa. Public Heal Pract. 2021 Nov;2:100076.

77. Penn-Kekana L. COVID-19 and MNCH: Beyond the models, what are we hearing from countries? 2020.

78. Physicians Across Continents. Community surveillance Report May 20 - Syria. 2020.

79. Rahimov BB, Singh K, Bazar C. Integrating screening for acute malnutrition into the vitamin A supplementation campaign in the Rohingya camps during the pandemic. Field Exchange - Emergency Nutrition Network ENN. 2020;(63):61–3.

80. Ramoni R. How COVID-19 Is Affecting Antenatal Care. Daily Trust. 2020 Jun;

81. Reuters. Child malnutrition at record highs in parts of Yemen: U.N. survey. Reuters. 2020 Oct;

82. Richards K, Abdi M, Stephenson H, Northcote C, Mathieson K. Nutrition Critical: Why we must act now to tackle child malnutrition. London; 2020.

83. Riley T, Sully E, Ahmed Z, Biddlecom A. Estimates of the Potential Impact of the COVID-19 Pandemic on Sexual and Reproductive Health In Low-and Middle-Income Countries. Int Perspect Sex Reprod Health. 2020;46.

84. Roberts L. Why measles deaths are surging — and coronavirus could make it worse. Nature. 2020 Apr;446–7.

85. Roberts L. Pandemic brings mass vaccinations to a halt. Science (80- ). 2020;368:116–7.

86. Save the Children. 60 Million Children Across Eight of the Biggest Humanitarian Crises Need Help to Survive this Year, Warns Save the Children. 2021 Jan;

87. Save the Children. Impact of Covid19 Outbreak on women and children: Save the Children Somalia Multi Sector Study. 2020.

88. Save the Children. YEMEN: Tens of thousands of severely malnourished children are left without treatment since March. 2020 Jun;

89. Stein D, Ward K, Cantelmo C. Estimating the Potential Impact of COVID-19 on Mothers and Newborns in Low- and Middle-Income Countries. Health Policy Plus. 2020.

90. The Global Fund. Mitigating the impact of COVID-19 on countries affected by HIV, tuberculosis and malaria. Geneva; 2020.

91. UN Country Office Syria. COVID-19 Socio-Economic Impact Assessment. 2020.

92. UNFPA. SRH Programme in NWS 2020 during the COVID-19 pandemic. 2021.

93. UNICEF. UNICEF Bangladesh Country Office. COVID-19 Response Monthly Report 17. 2020.

94. UNICEF. Trends and implications of Covid in the health sector - Somalia. 2020.

95. UNICEF UK. Malnutrition surges among young children in Yemen as conditions worsen. UNICEF UK. 2020 Oct;

96. UNICEF. Analysis of Continuity of Critical Health and Nutrition Services - Zimbabwe. 2020.

97. Walker D, Chandir S. COVID-19’s lost generation of unvaccinated children. 2021;

98. Khamala Wangamati C, Sundby J. The ramifications of COVID-19 on maternal health in Kenya, Sexual and Reproductive Health Matters. 2020;28(1).

99. West K. Collateral impacts of COVID-19 on non-COVID Health - An Update. 2020.

100. World Health Organization. The potential impact of health service disruptions on the burden of malaria: a modelling analysis for countries in Sub-Saharan Africa. Gene; 2020.

101. World Vision. A Double-Edged Sword. Protection Risks Facing Venezuelan Children During the COVID-19 Pandemic. 2020.

102. The Lancet Global Health. Food insecurity will be the sting in the tail of COVID-19. Vol. 8, The Lancet Global Health. Elsevier Ltd; 2020. p. e737.
